# Nonviral targeted mRNA delivery: principles, progresses, and challenges

**DOI:** 10.1002/mco2.70035

**Published:** 2025-01-02

**Authors:** Xi He, Guohong Li, Letao Huang, Haixing Shi, Sha Zhong, Siyu Zhao, Xiangyu Jiao, Jinxiu Xin, Xiaoling Yin, Shengbin Liu, Zhongshan He, Mengran Guo, Chunli Yang, Zhaohui Jin, Jun Guo, Xiangrong Song

**Affiliations:** ^1^ Department of Critical Care Medicine State Key Laboratory of Biotherapy West China Hospital Sichuan University Chengdu Sichuan China; ^2^ State Key Laboratory of Quality Research in Chinese Medicine Macau Institute for Applied Research in Medicine and Health Macau University of Science and Technology Taipa Macau China

**Keywords:** delivery obstacles, mRNA therapeutics, nonviral delivery, targeted delivery systems

## Abstract

Messenger RNA (mRNA) therapeutics have garnered considerable attention due to their remarkable efficacy in the treatment of various diseases. The COVID‐19 mRNA vaccine and RSV mRNA vaccine have been approved on the market. Due to the inherent nuclease‐instability and negative charge of mRNA, delivery systems are developed to protect the mRNA from degradation and facilitate its crossing cell membrane to express functional proteins or peptides in the cytoplasm. However, the deficiency in transfection efficiency and targeted biological distribution are still the major challenges for the mRNA delivery systems. In this review, we first described the physiological barriers in the process of mRNA delivery and then discussed the design approach and recent advances in mRNA delivery systems with an emphasis on their tissue/cell‐targeted abilities. Finally, we pointed out the existing challenges and future directions with deep insights into the design of efficient mRNA delivery systems. We believe that a high‐precision targeted delivery system can greatly improve the therapeutic effects and bio‐safety of mRNA therapeutics and accelerate their clinical transformations. This review may provide a new direction for the design of mRNA delivery systems and serve as a useful guide for researchers who are looking for a suitable mRNA delivery system.

## Graphical abstract

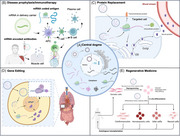

## 1 INTRODUCTION

Messenger RNA (mRNA), as a bridge between genes and proteins, was first discovered and studied in some papers from 1947 to 1961.^1^ Subsequently, the structural and functional aspects of mRNA were explored, leading to the development of in vitro transcribed (IVT) mRNA by the 1980s.^2^ The first animal experiments conducted in 1990 provided evidence that IVT mRNA could be translated in vivo.^3^ Despite significant findings since then, no substantial progress has been made in the application of mRNA‐based therapeutics. Consequently, it is necessary to promote further scientific and technological research to overcome the barriers associated with mRNA, such as instability, immunogenicity, and limited cellular uptake.^4^

**FIGURE 1:**
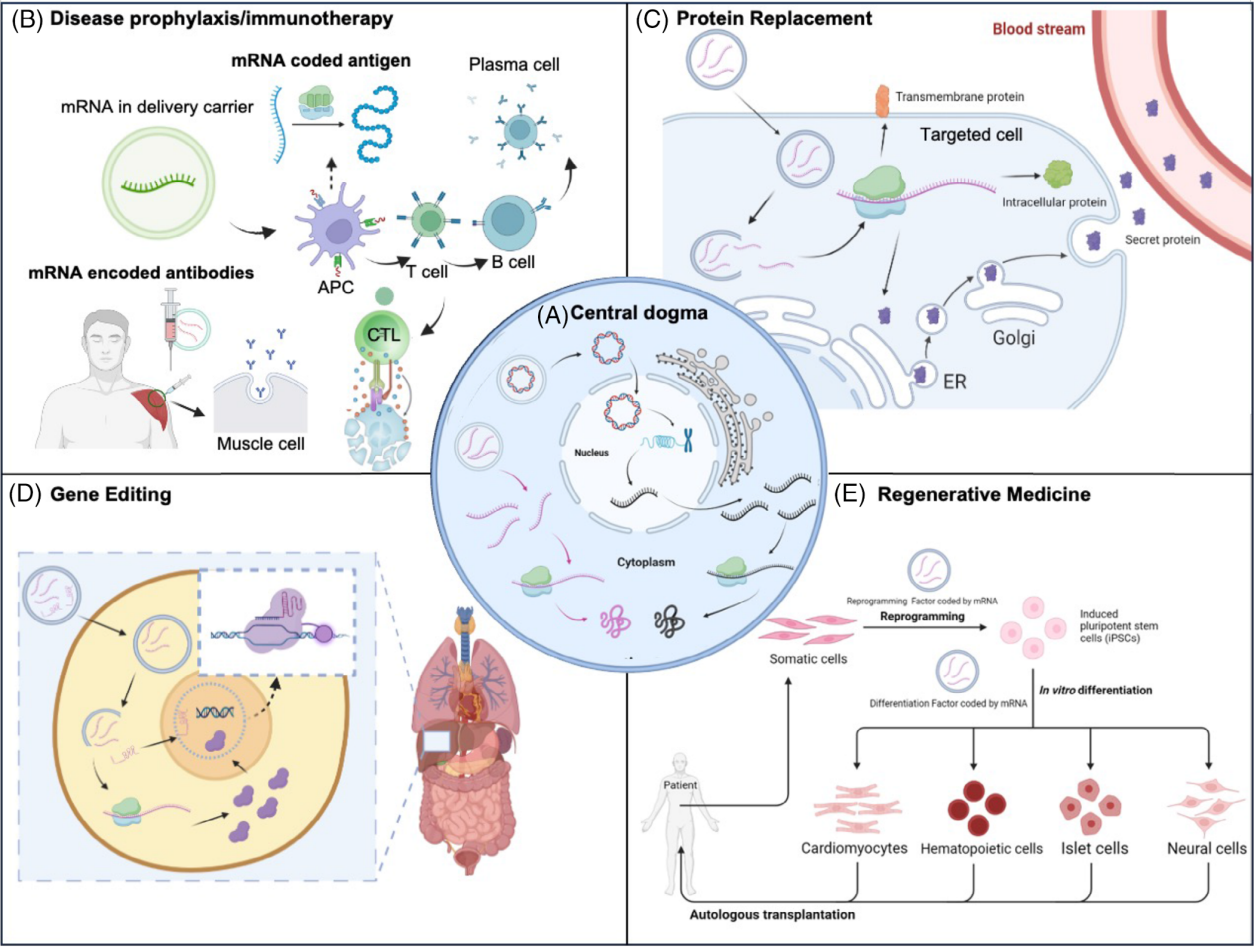
Schematic illustration of mRNA therapeutics. (A) The central dogma. mRNA therapeutics work through the expression of functional proteins or peptides in cytoplasm. (B) For disease prophylaxis or immunotherapy, mRNA encoding an antigen is internalized by somatic cells (e.g., muscle cells) or antigen‐presenting cells (APCs) after intramuscular injection. Then, antigens expressed in the cytoplasm are degraded by proteasomes, and then effector cells (e.g., T cells and B cells) are activated to detect and eradicate pathogens directly. The therapeutic antibodies can also be produced by mRNA for passive immunity. (C) mRNA has been used for protein replacement therapy, encoding transmembrane, intracellular, and secreted proteins. (D) mRNA can be applied to encode the Cas9 protein for gene editing in vivo. (E) mRNA encoding reprogramming factors can reprogram cells into induced pluripotent stem cells in vitro, which can differentiate into desired functional cells for tissue regeneration.

mRNA therapeutics work through mRNA‐encoded functional proteins and have shown great potential in various applications, including disease prophylaxis/immunotherapy, protein replacement, gene editing, and cellular reprogramming (Figure 1). mRNA‐based medicines offer several advantages over other nucleic acid‐based therapies. The protein expression of mRNA does not depend on the function of the nucleus like DNA does. Once mRNA reaches the cytoplasm, proteins can be efficiently produced even in nondividing cells. In addition, mRNA is generally regarded as a safer alternative to DNA because it cannot be integrated into the host genome, which makes it free from the risk of oncogenic effects and insertable mutations. While the protein encoded by IVT mRNA can be expressed rapidly, usually as soon as 1 h after transfection and peaking at 5–7 h.^5^ Moreover, compared with other traditional drugs, the greatest strength of mRNA‐based medicines is their remarkably swift development. For example, the COVID‐19 mRNA vaccine of Moderna was designed within just 2 days after obtaining the genetic sequence of the novel coronavirus in January 2020. Subsequently, they conducted clinical trials over the following months and received emergency use authorization in December 2020. This means the entire process took approximately less than a year from the viral genetic sequence to authorization. This speed surpassed that of any previous vaccine development, showcasing the great potential of mRNA‐based therapeutics.

Despite the advantages mentioned above of mRNA therapeutics, their practical implementation could be improved by several significant challenges. First, the single‐stranded mRNA can easily be degraded by ribonuclease (RNase), which is abundant in the environment and the human body. On the other hand, mRNA must reach the cytoplasm and produce sufficient proteins of interest for treatment, but their negative charges prevent them from crossing the same negatively charged cell membrane alone. The protective measures are required to ensure the effective application of mRNA‐based drugs.

A large number of delivery systems have been developed and applied for mRNA delivery, which can be divided into two types: viral and nonviral vectors.^6^ Viral vectors provide high transfection efficiency and sustainable expression; however, they have genetic toxicity, poor targeting potential, and very high costs. On the contrary, nonviral vectors are relatively less toxic, capable of transferring large quantities of mRNA, and easy to prepare, while they do not trigger unwanted immune reactions. The commonly applied nonviral vectors include lipid nanoparticles (LNPs), positively charged polymers, peptide/protein‐based systems, and so on. Among these vectors, LNPs were the most successful delivery system and have been applied in the approved mRNA vaccines (COVID‐19: BNT162b2/BioNTech and mRNA‐1273/Moderna, RSV: mRNA‐1345/Moderna). However, commercial LNPs all deliver mRNA to the liver, and the workplaces of mRNA therapeutics are quite different for the treatments of various diseases. It presents a high demand for specific tissue/cell‐targeted delivery, which may greatly improve the therapeutic effects and bio‐safety of mRNA drugs.

In this review, we first described the main obstacles faced during the process of mRNA delivery. The fundamental concepts and essential factors that needed to be considered for designing efficient mRNA delivery systems were outlined. The carriers used for mRNA delivery were summarized with an emphasis on LNPs. Then, we focused on the recently advanced developments in tissue/cell‐targeted delivery systems applied in mRNA‐based therapeutics in the preclinical and clinical. Finally, we generally discussed the challenges and offered our perspectives on the future developments of targeted mRNA‐delivery systems and mRNA therapeutics. We hope that this review can inspire continued research into targeting strategies of efficient mRNA delivery systems to realize the potential of mRNA therapeutics.

## 2 OBSTACLES OF mRNA DELIVERY IN VIVO

### 2.1 Degradation by the ubiquitous RNase

**FIGURE 2:**
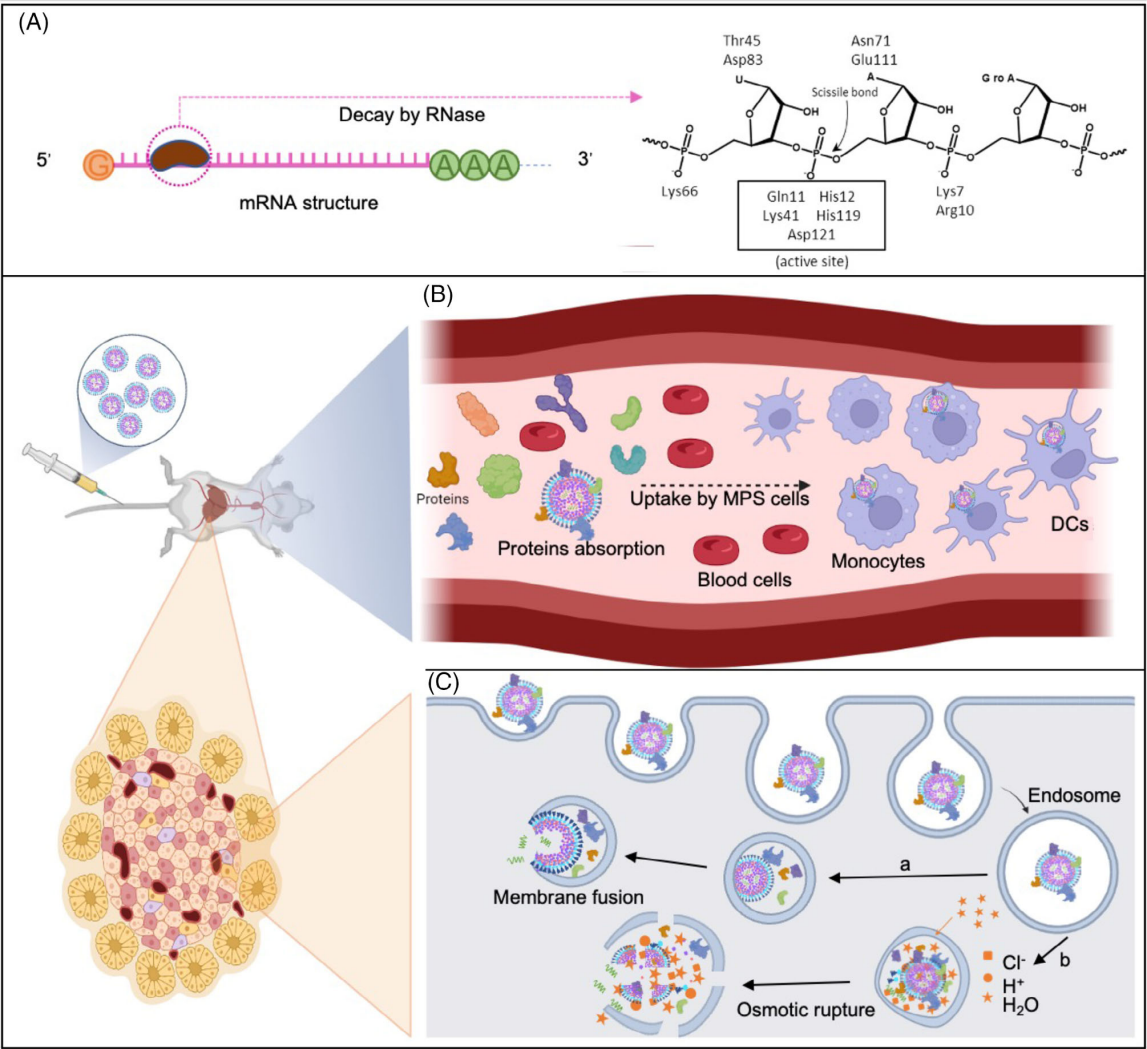
The in vivo obstacles encountered in the mRNA delivery process. (A) The phosphodiester bonds in the structure of mRNA are highly susceptible to degradation by RNA enzymes in the physiological environment. (B) mRNA‐containing formulations, such as LNPs, will rapidly get covered with various circulatory proteins and form the protein corona after intravenous administration. It causes the nanoparticles to be easily captured by MPS (e.g., macrophages in the liver) and cleared out of the body. (C) Furthermore, a portion of LNPs can enter target cells. The encapsulating mRNA can be released into the cytoplasm via (a) membrane fusion and (b) proton sponge effect‐mediated lysosomal escape.

The first challenge encountered in the delivery process is the RNase existing everywhere, which mediates mRNA degradation by converting the 5′‐triphosphate to a monophosphate^7^ (Figure 2A). To address this issue, the carrier should block the recognition between nucleases and ligands or provide an enzyme‐free environment. One approach is to bundle mRNA strands through the hybridization of RNA oligonucleotide linkers and prepare the mRNA nano‐assemblies (R‐NAs), which enhances stability toward RNase while preserving translational activity.^8^ However, the protein expression level mediated by R‐NAs is only one‐third of that of naive mRNA delivered with a commonly used lipid‐based transfection reagent. Developing delivery systems may be a more efficient direction to promote the mRNA expression in vivo. Up to now, the approved RNA‐based therapeutics such as Patisiran (siRNA), BNT162b2 (mRNA), and Spikevax (mRNA), all used LNPs as delivery systems to protect RNA from nucleases.^9^, ^10^, ^11^

### 2.2 Rapid clearance by the reticuloendothelial system

The clearance by the mononuclear phagocyte system (MPS) is another obstacle for mRNA therapeutics in vivo.^12^ A major fraction of nanoparticles distributes in the organs with an abundant accumulation of macrophages (e.g., lung, liver, and spleen) after intravenous administration (Figure 2B).^13^, ^14^, ^15^, ^16^, ^17^ Nanoparticles in the bloodstream will be combined with plasma proteins to form the proteins corona due to the electrostatic interaction, which makes them more prone to be recognized and cleared by phagocytic cells from the circulation, leading to the suboptimal therapeutic effects.^18^ The surface of the nanoparticles can be modified by using hydrophilic moieties, such as polyethylene glycol (PEG), which helps them to escape from MPS uptake, thus increasing time in the bloodstream and preventing unnecessary exposure to normal tissues.^13^ Moreover, the Li group^19^ labeled liposomes with a CD47‐derived “self” peptide, which was a putative marker giving phagocytes a “don't‐eat‐me” signal. The CD47‐labeled liposomes could escape the phagocytosis and clearance by phagocytes, improving their in vivo circulation time.^19^

### 2.3 Endosome escape

After escaping clearance by MPS, mRNA loaded by delivery systems is successfully internalized by cells, but still needs to escape from endosomes and be released into the cytoplasm, where translation occurs (Figure 2C). When carriers are internalized via endocytosis or pinocytosis, many imaging studies have demonstrated that nucleic acids are primarily accumulated inside endosomes or lysosomes with only a tiny portion released into the cytoplasm.^20^, ^21^, ^22^, ^23^ Compared with interstitial fluid or cytoplasm, the relative acidity of endosomes will lead to the significant degradation of nanoparticles encapsulated with mRNA, and they must be able to leave the endosome named endosome escape.^24^ Several mechanisms have been proposed for endosomal escape mediated by delivery systems.

#### 2.3.1 Osmotic rupture

The most common one is osmotic rupture, also known as the “proton sponge effect.” The pH will continuously decrease because of the influx of protons during endosomal maturation. When nanoparticles with pH buffering capacity enter endosomes, they start to resist the decrease of the pH inside the endosomes, which leads to a further influx of proton for lowering the pH and is followed by an influx of Cl^−^ and subsequently H_2_O. This results in high osmotic pressure, causing the swelling and rupture of endosomes.^20^, ^25^, ^26^ This mechanism is commonly observed in polyplexes (e.g., polyethyleneimine [PEI])^27^ and LNPs formulated with ionizable lipids,^28^ which are neutral at physiological pH and change to positive charge after being protonated in the endosomes. Thus, the endosomes can be disrupted by LNPs via the proton sponge effect, and mRNA can be released into the cytoplasm to work out.

#### 2.3.2 Membrane fusion

Endosomal escape can be achieved by the fusion of nanoparticles with the endosomal membrane. The fusion process destabilizes the membrane and allows the endosomal cargo to be released into the cytoplasm. This mechanism of endosome escaping is usually seen in the cases of viruses. As for the nonviral delivery system, the cationic lipids in lipoplex can complex with the anionic mRNA to form an inverted hexagonal structure (H_II_), and then the cargo encapsulated in lipid tubules leads to more effective release through fusion.^29^ In addition, the helper lipid dioleoylphosphatidylethanolamine (DOPE) has been proven to increase the transfection efficiency of lipoplex by promoting the H_II_ formation.^30^

#### 2.3.3 Membrane destabilization

Membrane destabilization may be mediated by several factors. Some peptides (e.g., perforin protein) may insert themselves between the phospholipid chains and result in their rearrangement into pore‐like structures.^31^ Then, it selectively binds to the opened pore structure lipid molecules and keeps the pore open to allow endosomal escape.^32^ Another interesting mechanism for endosomal escape is the photochemical lysis, which is called photochemical internalization. The cargos can be released into the cytoplasm with the help of exposure to light. Photosensitizers are commonly modified or encapsulated in the delivery system and locally accumulate in the endo and lysosomal membranes.^33^ When exposed to light, these photosensitizers can induce the generation of singlet oxygen species that break the endosomal or lysosomal membranes to release the cargo.

## 3 THE DESIGN STRATEGIES OF mRNA DELIVERY SYSTEMS

### 3.1 The mRNA‐loading mechanisms

**FIGURE 3:**
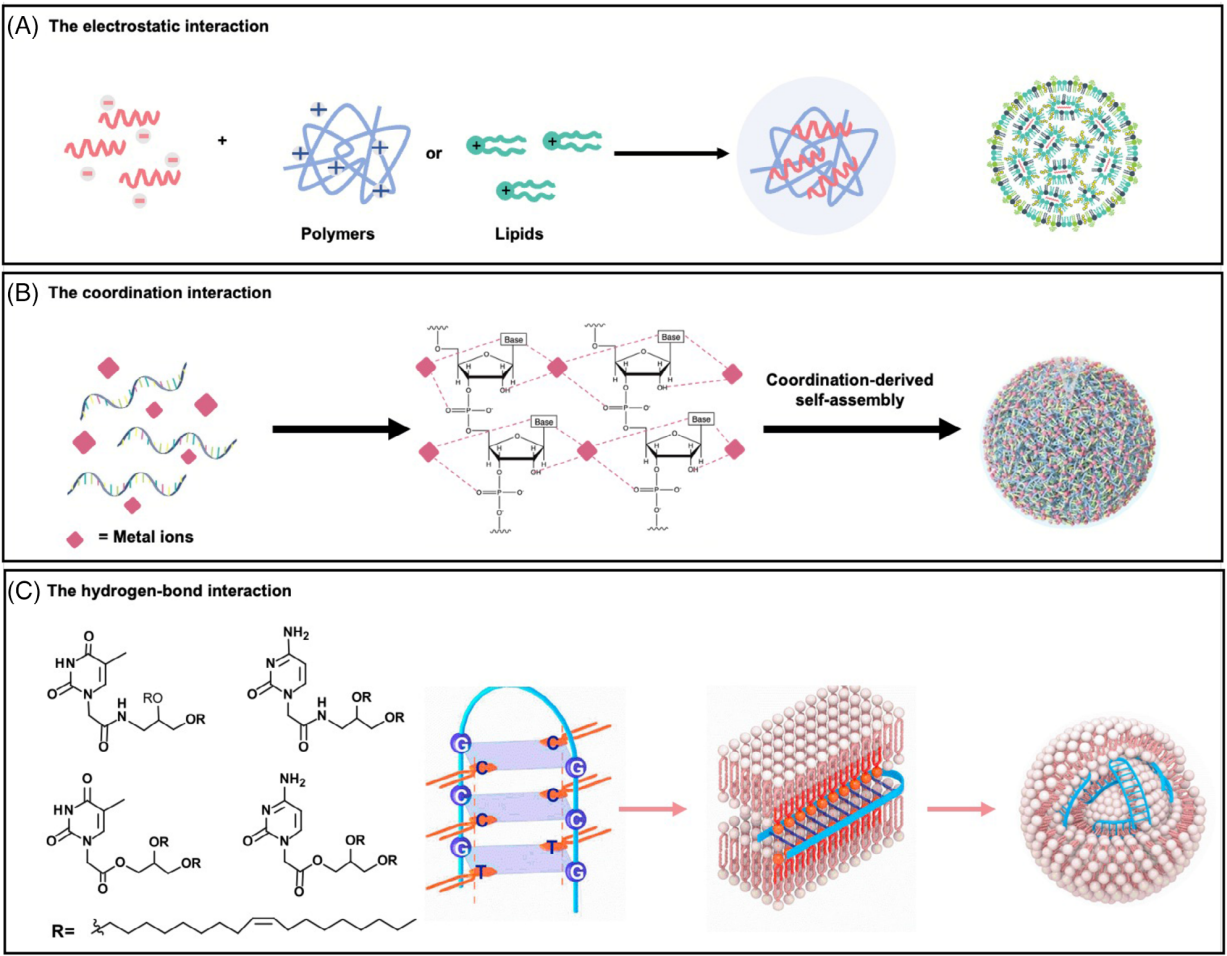
The schematic illustration of mRNA‐loading mechanisms. (A) The stable nanoparticles can be formed with negatively charged mRNA and positively charged polymers or lipids. (B) The mRNA can coordinate with metal ions and self‐assembly form spherical nanoparticles while retaining the integrity and biological function of RNA. (C) Furthermore, a novel class of nucleobase‐lipids termed DXBAs enables them to bind to oligonucleotides via the H‐bonding (principle of complementary base pairing) and p‐p stacking with reduced toxicity in vitro and in vivo.

Numerous delivery systems have been developed to enhance the delivery of mRNA, including lipid‐based systems, polymer‐based systems, polypeptide‐based systems, and various other delivery systems. It is noteworthy that distinct carrier materials may exhibit differential capacities for mRNA loading, thereby influencing their overall efficacy. The loading of mRNA into delivery systems may involve a range of mechanisms, including electrostatic interaction, coordination interaction, and hydrogen‐bond interaction (Figure 3). These diverse molecular interactions play a crucial role in facilitating the successful loading of mRNA. Recognizing the significance of mRNA loading holds immense potential for designing and developing efficient delivery systems.

#### 3.1.1 Electrostatic interaction

mRNA is a negatively charged biopolymer, carrying one charge per phosphodiester bond, which can electrostatically interact with the cations to load mRNA.^34^ Interestingly, cation concentration and identity can critically influence global and local structure. On the other hand, cationic lipids commonly present alkylated quaternary ammonium groups that retain their permanent positive charges regardless of pH.^13^, ^35^ However, it may lead to the possible cytotoxicity, limiting their application.^36^

#### 3.1.2 Coordination interaction

In addition, amino nitrogen (p*K*_a _= 4),^34^ phosphate oxygens,^34^ and keto oxygens^37^, ^38^, ^39^ in RNA are electron‐rich groups that can coordinate metal ions. Recently, Li et al.^40^ developed a Fe^2+^ coordination‐induced self‐assembly methodology for the synthesis of Fe‐DNA nanospheres, laying a foundation for nucleic acid coordination nanomaterials. However, when mRNA is under conditions that near neutral pH and the presence of alkali metals and alkali‐earth metals inside cells, the inherent chemical instability of its RNA phosphodiester bonds will be a risk to the integrity of mRNA.^41^ The proximity of the adjacent 2′‐hydroxyl group to the phosphorus center of each internucleotide linkage permits facile transesterification to occur, particularly under strongly acidic or fundamental conditions. Although some metal ions may alter electron density and proton transfer, accelerating mRNA degradation, specific metal ions may be suitable for mRNA delivery, which depends on the coordination site, coordination way, or affinity.^39^ The two kinds of purine and the cytosine residues contain imidazole‐ and pyridine‐type nitrogen, which are well‐suited for combination with divalent metal‐ion (M^2+^).^39^ For example, it was reported that Zn^2+^‐driven RNA self‐assembly forming spherical nanoparticles can load GFP‐mRNA and make protein expressed in HeLa cells.^42^

#### 3.1.3 Hydrogen‐bond interaction

The bases on the mRNA provide the ability for hydrogen bonding or π–π stacking. Lipid derivatives of nucleoside analogs have received much attention from researchers due to their potential for effective gene delivery, among which Yang's group has done a lot of work based on nucleoside analogs. Yang's group designed a cationic lipid CLD with independent intellectual property rights, which uses an amide bond and amino group as the hydrophilic head and an unsaturated alkyl chain as the hydrophobic tail. The two chains are connected by a disulfide bond, which can be cleaved under the condition of cytokines reduction to reduce lipid toxicity.^43^, ^44^, ^45^, ^46^ On this basis, the team designed new nucleotide lipids (thymine nucleoside analogues^43^ and cytosine nucleoside analogues^44^) through hydrogen bonding and π‐π stacking interaction and oligonucleotide binding, mixed with CLD, capable of delivering a variety of nucleic acid drugs with low toxicity in vivo and in vitro. The cytosine nucleoside analogs lipids DNCA/CLD^45^ were used to deliver SARS‐CoV‐2 RBD antigen‐encoded mRNA in mice, which could generate specific antibodies and neutralizing antibodies (NT50 could reach ∼1:10591). In inoculated mice with influenza A virus HA mRNA vaccine to express HA protein and produce neutralizing antibody, after influenza virus challenge (lethal dose) for 14 days, the survival rate of immunized mice was 100%, while unimmunized mice were 20%. DNCA/CLD containing cyclic dinucleotides has a good immunotherapeutic effect on breast cancer, melanoma, and lung cancer.^46^

In addition to the loading mechanisms mentioned above, the cell‐derived vehicles can encapsulate the mRNA introduced into cell ex vivo,^47^ such as red blood cells (RBCs).^48^ The extracellular vesicle (EV) is the other system that directly encapsulates mRNA, including extracellular vesicles (EVs)^47^ and a novel vehicle named SEND.^49^, ^50^

### 3.2 Nonviral mRNA delivery systems

mRNA delivery vectors can be broadly classified into two categories: viral vectors and nonviral vectors. As for the viral vectors, the modified viruses such as retroviruses, lentiviruses, adenoviruses, and adeno‐associated viruses have been used to deliver genes. Although they have substantially advanced the field of gene therapy, there are still several limitations, including immunogenicity,^51^ carcinogenesis,^52^ restricted cargo packaging capacity,^53^ and difficulty of scaled production.^54^ On the contrary, nonviral systems showed great potential to address many of these issues, particularly regarding safety.

#### 3.2.1 Lipid‐based systems

Among the nonviral nucleic acid vectors, lipid‐based systems (e.g., LNPs, liposome, lipoplex) have emerged as the most employed approach.^55^ DOTMA (N‐(1‐(2,3‐dioleyloxy)propyl)‐N, N, N‐trimethylammonium chloride) was the first synthetic cationic lipid utilized to deliver IVT mRNA. The vehicle loading the mRNA successfully transfected in humans, rats, mice, Xenopus (frog), and drosophila cells in vitro.^55^, ^56^ DOTAP (1,2‐dioleoyl‐3‐trimethylammonium‐propane), derived from DOTMA, has been widely studied due to its lower manufacturing cost and higher delivery efficiency.^55^, ^57^ Zwitterionic lipid DOPE was then applied with DOTAP to increase the transfection efficiency.^58^ The cationic lipids can also be used to prepare cationic nano‐emulsions (CNEs), which are prepared by homogenizing the aqueous/oil phases containing excipients and can subsequently be complexed with mRNA.^55^, ^59^ A self‐amplifying RNA (saRNA) vaccine that expresses the type I envelope antigen of human immunodeficiency virus (HIV) was formulated into CNEs and induced potent immune responses in rhesus macaques.^60^

With the development of microfluidics, LNPs can be efficiently prepared with cationic/ionizable lipids, electrically neutral phospholipids, cholesterol, and PEGylated‐lipids, and it can much enhance the encapsulation and transfection efficiency of mRNA.^61^ Notably, LNPs have gained significant recognition and have been at the forefront of clinical developments. In fact, the United States Food and Drug Administration has granted approval for three drugs, namely Patisiran, BNT162b2, and mRNA‐1273, all of which are developed based on LNPs. While the different components in LNPs have specific functions: cationic/ionizable lipids provide a positive charge to combine with negatively charged mRNA and help their endosomes escape; the phospholipids (e.g., DOPE, DSPC, PC, PS) and cholesterol can help the formation and stability of LNPs structures; the PEGylated‐lipids (e.g., DMG‐PEG2000, ALC‐0159) can prevent the nonspecific binding from the plasma proteins, prolonging the in vivo circulating half‐life of LNPs.

**FIGURE 4:**
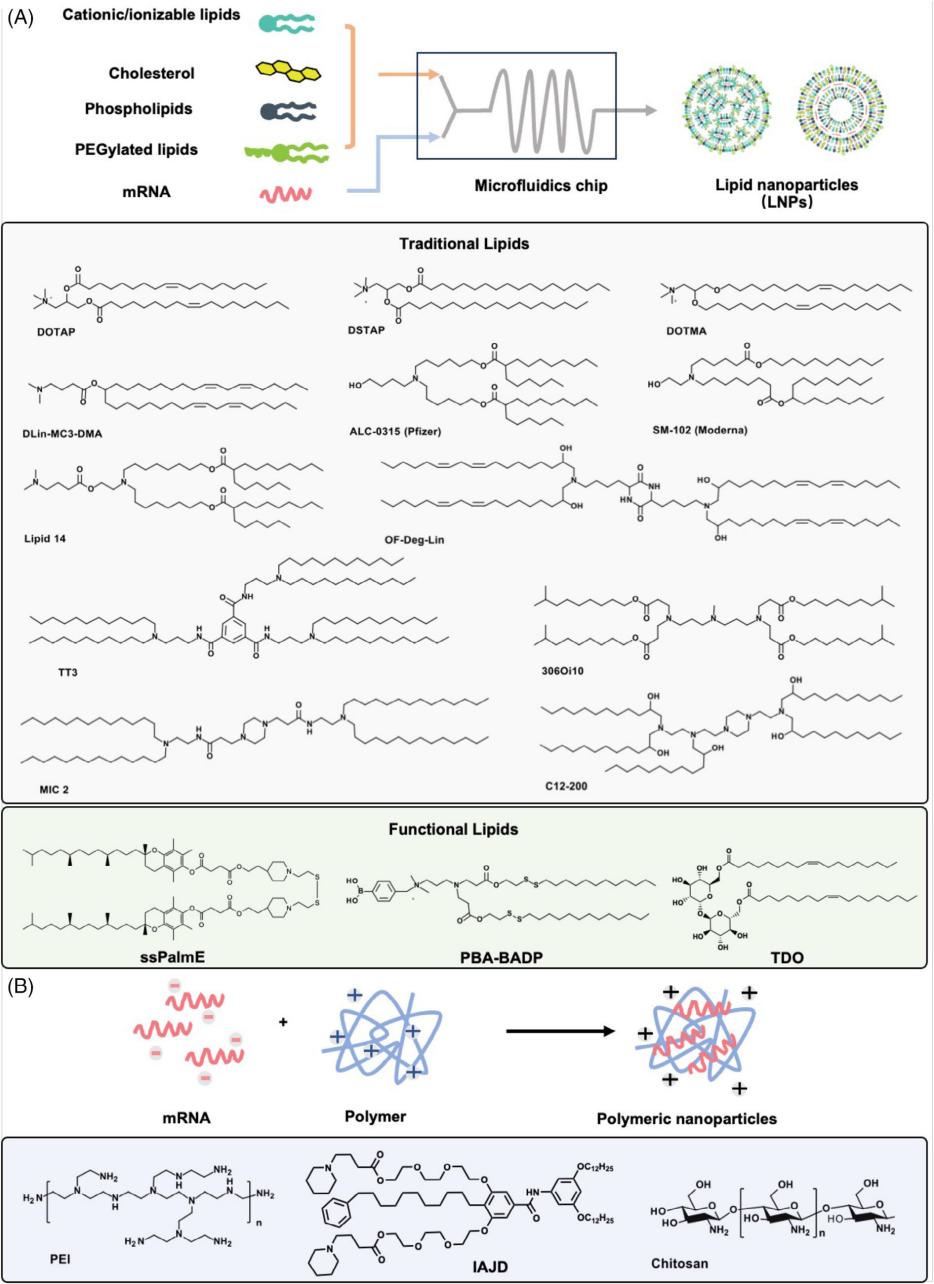
The common structures of the delivery systems. (A) Lipid nanoparticles (LNPs) are typically prepared by microfluidics with traditional (cationic or ionizable) lipids or functional lipids, cholesterol, and helper lipids. (B) The polymer‐based vectors, including polyethyleneimine (PEI),^74^ ionizable amphiphilic Janus dendrimer (IAJD),^81^ and chitosan, can encapsulate mRNA to form stable nanoparticles via simple mixing.

Among these components in LNPs, cationic/ionizable lipids have the greatest influence on their mRNA delivery capacity,^47^ especially on their chemical structures (Figure 4A). Cationic lipids (e.g., DOTAP, DSTAP, DOTMA) commonly contain the alkylated quaternary ammonium salt groups. These lipids can maintain cationic properties regardless of pH changes in the physiological environment. However, some cationic lipids can cause cytotoxicity due to their permanent positive charge.^62^, ^63^ For example, cationic lipids might reduce mitosis in cells, form vacuoles in the cellular cytoplasm, and cause detrimental effects on key cellular proteins.^64^ It was also reported that amphiphiles with quaternary ammonium head groups are more toxic than those with tertiary amine head groups.^65^ Ionizable lipids (e.g., DLin‐MC3‐DMA, ALC‐0315, SM‐102) are neutrally charged in a physiological environment (pH ∼7.4), which can interact less with anionic blood cells and proteins, prolonging the circulation of LNPs in vivo. Once in a low pH environment, such as an endosome within a cell, ionizable lipids will be rapidly protonated and positively charged.^66^ This process may lead to instability of the endosome membrane, promoting endosomal escape of LNPs and releasing mRNA into the cytoplasm.^67^ Therefore, compared with cationic lipids, ionizable lipids enhance the mRNA delivery efficiency of LNPs and further improve their potential toxicity.^68^

Efforts in LNPs development include identifying new lipids, optimizing lipid components, and modifying LNPs with functional moieties. A traditional strategy to design new lipids is to chemically combine with an amino head, a hydrophobic tail, and a linker between the head and tail, and altering any of these three parts can change the structures and properties of the lipids.^69^ On the other hand, some functional lipids were developed to achieve enhanced mRNA delivery and therapeutic effects. Akita et al.^70^ constructed a novel lipid with a vitamin E scaffold (ssPalmE), which could act as an adjuvant to stimulate type I interferon signaling and promote antitumor immunotherapeutic effects. While Wang group^71^ reported a cationic lipid PBA–BADP conjugated with phenylboronic acid that enabled a specific interaction with sialic acid overexpressed on the surface of cancer cells to enhance the cellular internalization of PBA–BADP/mRNA NPs. In addition to the mRNA transfection efficiency, the potential toxicity and clinical safety of LNPs have been paid much more attention in recent years. Thus, Bang and coworkers^72^ designed a novel trehalose glycolipid TDO that was intended to promote a stable formulation of particles via hydrogen bonding and reduce the toxicity caused by ionizable lipids, maintaining their mRNA delivery efficacy.

#### 3.2.2 Polymeric systems

Cationic/ionizable polymers (Figure 4B), including PEI, dendrimers, and chitosan, have attracted more attention and have been used for mRNA delivery due to their great flexibility in structural modification.^73^ PEI is the most widely investigated polymer for nucleic acid delivery. Because of the abundant amino groups, PEI can provide a high density of positive charges for mRNA encapsulation and show excellent endosomal escape caused by the “proton sponge” mechanism.^74^ However, the efficiency–toxicity correlation is unsatisfactory in PEI‐mediated mRNA delivery. The PEIs with high molecular weights show considerable delivery efficiency but suffer from serious cytotoxicity, while ones with low molecular weight are minimally toxic but with low efficiency.^75^ Wang et al.^76^ designed a novel self‐assembled polymeric micelle based on vitamin E‐modified PEI1.8k (PVES) to deliver mRNA. PVES showed higher mRNA transfection efficiency and much lower cytotoxicity than PEI 25k (a gold standard in nucleic acid delivery). However, PEI is not degradable, and there are concerns regarding its toxicity, which limits its use for clinical applications. Thus, degradable cationic poly(β‐amino esters) (PBAEs) not only show high biodegradability because of the ester bonds but also have the advantages of facile synthesis and widespread availability of monomers^77^.^78^ Kim's team^79^ created a library of PBAE polymers by combining various amine monomers and acrylate monomers. Through screening this polymer library, the specific polymer nanoparticles (PNPs) were identified and could highly efficiently deliver mRNA in vivo with sustained mRNA expression for up to 2 weeks.^79^

The dendrimers are a kind of polymeric molecules branching out to form a spherical structure, in which poly(amidoamine) (PAMAM) is one of the most studied dendrimers. Shi et al.^80^ used a dendrimer–lipid hybrid system involving cationic PAMAM (generation 0) G0‐C14 to deliver phosphatase and tensin homolog deleted on chromosome 10 (PTEN) encoding mRNA to restore functional PTEN protein production, with consequent inhibition of tumor cell growth and induction of apoptosis. While Percec et al.^81^, ^82^, ^83^, ^84^, ^85^ developed a series of novel amphiphilic Janus dendrimers (IAJDs), which exhibited higher activity at low ionizable amine concentration for mRNA delivery.

In addition to these synthetic carriers, some naturally occurring polymers have also gained attention as carriers for nucleic acids due to their significant biocompatibility. Chitosan is a polysaccharide derived from chitin, which can interact electrostatically with nucleic acids. Chitosan is commonly used with other materials, such as poly (lactic‐co‐glycolic acid) (PLGA)^86^ and hyaluronic acids,^87^ to deliver mRNA for the improvement of stability and delivery.

The consistency of pharmaceutical drugs is necessary for successful clinical application, and most polymers may present limitations in clinical translation due to batch‐to‐batch variability. In fact, tiny differences in the molecular weight of polymers can significantly impact the transfection efficiency of mRNA in vivo. Thus, despite the abundant polymeric materials available, polymeric systems are still not as clinically advanced as lipid systems in mRNA‐based therapeutics.^55^

#### 3.2.3 Peptide or protein‐based systems

Positively charged peptides and proteins can condense negatively charged mRNA via electrostatic interaction and show good biocompatibility and degradability.

Peptides consist of at least two amino acids, and their inherent structural flexibility enables precise modulation of their cationic and endosomal dissolution properties.^88^ For example, the amphipathic peptides can help deliver mRNA into cells owing to their cationic or amphipathic amine groups, such as arginine, which can electrostatically bind to the mRNA and help to lysosome escape due to the proton sponge effect.^89^ Lam et al.^90^ reported a novel mRNA delivery vector, PEG12KL4, in which the synthetic cationic KL4 peptide (containing lysine and leucine) is attached to the hydrophilic PEG. PEG12KL4 has been proven an excellent mRNA transfection agent for pulmonary delivery. It could be well prepared for inhalable dry powder mRNA formulations with in vivo transfection efficiency.

Among the protein‐based delivery systems, protamine is a small, arginine‐rich, positively charged protein capable of packaging mRNA,^91^ which has already been applied in mRNA therapeutics. CureVac has developed a platform called RNAactive^®^ containing free and protamine‐complexed mRNAs that induce a balanced adaptive immune response as well as T cell‐mediated immunity.^92^ Based on this technology, CureVac has launched several projects, which are currently undergoing clinical trials.^93^, ^94^, ^95^ Recently, virus‐like particles (VLPs) comprise the major structural proteins of a virus needed to assemble a viral capsid but do not package the viral genomic material.^96^ They possess the delivery efficiency and targeting ability of viral vectors but obviate the risks of viral genome integration. Unti and Jaffrey^97^ applied VLP technology in packaging and in vivo delivery of circular mRNA, which could express circular mRNA with high efficiency in mammalian cells.

### 3.3 Considerations in the design of delivery systems

Understanding the mRNA loading mechanism and bio‐barriers helps us to identify the useful groups in carriers. However, the specific delivery of mRNA to tissues and cells in vivo remains a significant challenge.^98^Much research has reported the complicated interactions between nanoparticles and cells, as such information is key to rationally designing particles for biological applications, which may determine the cellular uptake leading to the specific accumulation. It was reported that the surface charge of LNPs at physiological pH can influence their plasma protein adsorption and in vivo behavior (e.g., tissue distribution).^14^, ^98^ A classic example is apolipoprotein E (ApoE), generally recognized as an endogenous ligand targeted to the liver.^99^ One explanation is that the neutral surface vehicle will absorb more ApoE, resulting in a stronger tendency to distribute in the liver.^100^ The dissociation constant (p*K*_a_) that determines the surface charge of LNPs at different pH is one of the important factors to consider when designing ionizable lipids. Other pieces of evidence also suggest that surface charge has an impact on the targeting ability of LNPs: the inclusion of neutral ionizable lipids enhanced liver targeting, anionic lipids resulted in retargeting of delivery to the spleen, and permanently cationic lipids bearing a quaternary ammonium headgroup specifically deliver mRNA to lung.^101^, ^102^ Interestingly, the above two nonliver targeting effects occur independently of ApoE, which suggests that there is a correlation between the components of protein corona and surface charges of nanoparticles, resulting in the differences in the tissue‐targeted ability^103^.^104^

A recent study found that LNPs prepared with the structurally similar ionizable lipids 306‐O12B and 306‐N16B showed slight negative charges without significant differences.^105^ However, 306‐O12B containing the ester linker led to liver targeting, while 306‐N16B with an amide linker tended to lung targeting. Compared with ester bonds, amides tend to combine with proteins via hydrogen bonds.^106^ Thus, the protein corona coating in 306‐N16B LNPs is completely different from that in 306‐O16B LNPs, which has been proven.^105^ These results demonstrated that the protein corona compositions of LNPs are not only associated with surface charges but also related to the chemical structures of ionizable lipids. However, a deep understanding of how the structure of ionizable lipids affects protein corona formation remains challenging, which will greatly help design efficient targeted mRNA delivery vectors. From another perspective, the participation of targeting ligands may endow the targeting ability with delivery systems and already have been applied in vivo,^107^, ^108^ which will be detailly discussed in the following section.

Another challenge for the efficient delivery vector is to control mRNA release.^109^ It should be noted that uptake is not always associated with transfection levels. Different chemical groups endow ionizable lipids with different properties, which may determine the mRNA‐transfected tissue. For example, Fenton et al.^110^ synthesized two structurally similar ionizable lipids OF‐02^109^ and OF‐Deg‐Lin,^108^ with a slight difference in their hydrophobic tails. OF‐02 with nondegradable tails and OF‐Deg‐Lin containing degradable ester linkages were formulated into LNPs and showed good mRNA transfection efficiency in vivo. Interestingly, following systemic administration, OF‐02 and OF‐Deg‐Lin LNPs mostly accumulated in the livers. However, degradable OF‐Deg‐Lin LNPs induced protein expression predominantly in the spleen, and nondegradable OF‐02 LNPs induced protein expression predominantly in the liver. This phenomenon could be attributed to the degradable property of OF‐Deg‐Lin. The degradable ester linkages are stable at physiological pH (∼7.4) but enzymatically hydrolyzed within tissues and cells. It is worth to be noticed that compared with the liver, the spleen contains a relatively low abundance of hydrolases.^111^ Thus, the LNPs are still transported to the liver but may be easily degraded before actively inducing protein expression. By contrast, these LNPs transported to the spleen can retain the ability to induce protein expression following uptake in the cells of the spleen. There is another example worth discussing. The hexagonal II (H_II_) phase formation of LNPs can promote membrane fusion and induce endosome escape to efficiently release mRNA into cytoplasm.^112^, ^113^ It was reported that lipids containing alkenyl^114^ or alkynyl groups^100^ could facilitate the H_II_ phase formation of LNPs. Siegwart et al.^115^ reported a series of multi‐tailed ionizable phospholipids (iPhos) capable of delivering mRNA or mRNA/single‐guide RNA for gene editing in vivo. With further investigation, it could be found that iPhos adopt a cone shape in endosomal acidic environments to facilitate membrane hexagonal transformation and subsequent mRNA release from endosomes.

Compared with mRNA, the delivery systems for siRNA have been accumulating research for quite a long time.^116^, ^117^ Thus, a series of mRNA delivery materials were derived from siRNA delivery materials. 1,2‐dilinoleyloxy‐N, N‐dimethyl‐3‐aminopropane, as an efficient siRNA delivery material,^118^ has been used for the saRNA delivery.^119^ In the realm of siRNA delivery, rational design strategies have been employed to enhance the delivery of synthetic lipids by systematically altering structural elements in the lipid head, linker, and tail regions. Based on these systematic approaches, a plethora of novel analogs have been yielded, including DLin‐KC2‐DMA,^120^ DLin‐MC3‐DMA,^114^ and L319.^121^ It may be beneficial to develop a new mRNA delivery vector based on the design strategy of the siRNA delivery vector. Additionally, many mRNA delivery systems have already been developed through the methods of combinatorial chemistry and library screening.^122^, ^123^, ^124^, ^125^, ^126^, ^127^, ^128^

### 3.4 The clinical applications of nonviral mRNA delivery systems

Up to now, a total of 2517 mRNA‐related registered clinical trials can be retrieved from the website clinicaltrail.gov, of which 120 are in the early phase I, 767 are in clinical phase I, 758 are in clinical phase II, 610 are in clinical phase III, and 487 are in clinical phage IV. The indications for these clinical trials include infectious diseases, tumors, metabolic diseases, viruses, cardiovascular diseases, and rare genetic diseases. Table 1 presents the clinical trials applied with nonviral delivery systems, while the clinical trials of COVID‐19 were excluded in the part of infectious diseases. It could be found that LNPs were the most widely used delivery system, demonstrating their applied potential in mRNA therapeutics.

**TABLE 1:** List of all conducted clinical trials for mRNA‐based treatments.

Applications	Delivery systems	Treatment principles	Product names	R&D institutions	Routes	Status	Trial numbers	Date
**Infectious diseases**								
Zika virus	LNPs	Immunotherapy	mRNA‐1325	ModernaTX, Inc.	IM	I	NCT03014089	2017
Zika virus	LNPs	Immunotherapy	mRNA‐1893	ModernaTX, Inc.	IM	II	NCT04917861	2021
Herpes Zoster	LNPs	Immunotherapy	IN001	Shenzhen Shenxin Biotechnology Co., Ltd	IM	I	NCT06375512	2024
HIV	LNPs	Immunotherapy	mRNA‐1574(BG505 MD39.3 mRNA/ BG505 MD39.3 gp151 mRNA/ BG505 MD39.3 gp151 CD4KO mRNA)	National Institute of Allergy and Infectious Diseases (NIAID) and ModernaTX, Inc.	IM	I	NCT05217641	2022
HIV	LNPs	Immunotherapy	Core‐g28v2/eOD‐GT8	International AIDS Vaccine Initiative and ModernaTX, Inc.	IM	I	NCT05001373	2021
HIV	DCs	Immunotherapy	mRNA‐transfected autologous dendritic cells	Massachusetts General Hospital	ID	I/II	NCT00833781	2009
HIV	Naked	Immunotherapy	iHIVARNA‐01	iHIVARNA consortium	Intranodal	II	NCT02888756	2016
Influenza	LNPs	Immunotherapy	MRT5421/MRT5424/ MRT5429	Sanofi Pasteur, a Sanofi Company	IM	I/II	NCT06361875	2024
Influenza	LNPs	Immunotherapy	sa‐mRNA vaccine	Seqirus and Arcturus Therapeutics	IM	I	NCT06028347	2023
Influenza	N/A	Immunotherapy	DCVC H1 HA mRNA	National Institute of Allergy and Infectious Diseases (NIAID)	IM	I	NCT05945485	2023
Influenza	LNPs	Immunotherapy	H1ssF_3928	National Institute of Allergy and Infectious Diseases (NIAID)	IM	I	NCT05755620	2023
Influenza	LNPs	Immunotherapy	H3 mRNA/LNP	Sanofi Pasteur, a Sanofi Company	IM	I	NCT05829356	2023
Influenza	LNPs	Immunotherapy	Influenza Hemagglutinin mRNA vaccine	Sanofi Pasteur, a Sanofi Company	IM	I	NCT06118151	2023
Influenza	LNPs	Immunotherapy	mRNA NA vaccine	Sanofi Pasteur, a Sanofi Company	IM	I	NCT05426174	2022
Influenza	LNPs	Immunotherapy	mRNA‐1010	ModernaTX, Inc.	IM	II	NCT05606965	2022
Influenza	LNPs	Immunotherapy	MRT5413	Sanofi Pasteur, a Sanofi Company	IM	I/II	NCT05650554	2022
SARS‐CoV‐2/seasonal influenza/ respiratory syncytial virus/ cytomegalovirus	LNPss	Immunotherapy	mRNA‐1273/mRNA‐1010/mRNA‐1345/mRNA‐1647	ModernaTX, Inc.	IM	I	NCT05397223	2022
Seasonal influenza	LNP	Immunotherapy	mRNA‐1010	ModernaTX, Inc.	SC	III	NCT05827978	2023
Influenza	N/A	Immunotherapy	GSK4382276A	GlaxoSmithKline	IM	II	NCT06431607	2024
Lyme disease	LNPs	Immunotherapy	mRNA‐1975 and mRNA‐1982	ModernaTX, Inc.	IM	I/II	NCT05975099	2023
Nipah virus infection	LNPs	Immunotherapy	mRNA ‐1215	ModernaTX, Inc. and National Institute of Allergy and Infectious Diseases (NIAID)	IM	I	NCT05398796	2022
Prevention of chikungunya virus infection	LNPs	Immunotherapy	mRNA‐1944	ModernaTX, Inc.	IV	I	NCT03829384	2019
Rabies	LNPs	Immunotherapy	CV7202	CureVac	IM	I	NCT03713086	2018
Rabies	Protamine	Immunotherapy	CV7201	CureVac	ID / IM	I	NCT02241135	2014
Respiratory syncytial virus infection	LNP	Immunotherapy	RSV/hMPV mRNA LNP	Sanofi Pasteur, a Sanofi Company	IM	I	NCT06237296	2024
Hepatitis B/healthy	ChAd	Immunotherapy	ChAdOx1‐HBV	Barinthus Biotherapeutics	IM	I	NCT04297917	2020
**Tumors**								
Melanoma	LNPs	Immunotherapy	mRNA‐4157	ModernaTX, Inc.	IV	II	NCT03897881	2019
Adult glioblastoma	DOTAP	Immunotherapy	RNA‐LPs	University of Florida	IV	I	NCT04573140	2024
Advanced malignant solid tumors	LPP	Immunotherapy	Neoantigen mRNA Personalised Cancer vaccine	Stemirna Therapeutics	SC	N/A	NCT05949775	2023
EBV‐positive advanced malignant tumors	LNPs	Immunotherapy	EBV mRNA vaccine	Westgene	IM	I	NCT05714748	2024
Solid tumor	LPP	Immunotherapy	SW1115C3	Stemirna Therapeutics	IM	I	NCT05198752	2022
Esophageal cancer/non small cell lung cancer	LPP	Immunotherapy	Personalized mRNA Tumor Vaccine	Stemirna Therapeutics	SC	N/A	NCT03908671	2019
Pulmonary osteosarcoma	LP	Immunotherapy	RNA‐LP vaccine	University of Florida	IV	I/II	NCT05660408	2022
Glioblastoma	LNPs	Immunotherapy	CV09050101(CVGBM)	CureVac	IM	I	NCT05938387	2023
Liver cancer	LNPs	Immunotherapy	HBV mRNA vaccine	Westgene	IM	I	NCT05738447	2023
Patients with advanced solid tumors	LNPs	Immunotherapy	IL‐12 mRNA	Cancer Institute and Hospital, Chinese Academy of Medical Sciences	Intratumoral injection	I	NCT05392699	2022
Relapsed/refractory solid tumor malignancies or lymphoma/ovarian cancer	LNPs	Immunotherapy	mRNA‐2416	ModernaTX, Inc.	Intratumoral injection	I/II	NCT03323398	2017
Acute myeloid leukemia	DCs	Immunotherapy	Antigen‐loaded cultured dendritic cells	University Hospital, Antwerp	ID	I	NCT00834002	2009
Acute myeloid leukemia/chronic myeloid leukemia/multiple myeloma	DCs	Immunotherapy	Dendritic cell vaccination	University Hospital, Antwerp	ID	II	NCT00965224	2009
Brain cancer/neoplasm metastases	DCs	Immunotherapy	Personalized cellular vaccine	Guangdong 999 Brain Hospital	N/A	I	NCT02808416	2016
Colorectal cancer/liver metastases	DCs	Immunotherapy	CEA‐loaded dendritic cell vaccine	Radboud University Medical Center	ID and IV	I/II	NCT00228189	2005
Esophagus cancer	DCs	Immunotherapy	Adenovirus‐transfected autologous DC vaccine plus CIK cells	Affiliated Hospital to Academy of Military Medical Sciences	N/A	I/II	NCT02693236	2016
Glioblastoma	DCs	Immunotherapy	Dendritic cell vaccine with mRNA from tumor stem cells	Oslo University Hospital	ID	I/II	NCT00846456	2009
Glioblastoma/renal cell carcinoma/sarcomas/ breast cancers/ malignant mesothelioma/colorectal tumors	DCs	Immunotherapy	Autologous dendritic cell vaccination	University Hospital, Antwerp	ID	I/II	NCT01291420	2011
Myelodysplastic syndromes/acute myeloid leukemia	DCs	Immunotherapy	Autologous dendritic cells electroporated with WT1 mRNA	University of Campinas, Brazil	Electroporation	I/II	NCT03083054	2017
Prostate cancer	DCs	Immunotherapy	Dendritic cell vaccine	Oslo University Hospital	ID	I/II	NCT01197625	2010
Prostatic neoplasms	DCs	Immunotherapy	mRNA transfected dendritic cell	Inge Marie Svane	ID	II	NCT01446731	2011
Recurrent central nervous system neoplasm	DCs	Immunotherapy	BTSC mRNA‐loaded DCs	John Sampson	N/A	I	NCT00890032	2009
Recurrent epithelial ovarian cancer	DCs	Immunotherapy	DC‐006 vaccine	Steinar Aamdal	N/A	I/II	NCT01334047	2011
Uveal melanoma	DCs	Immunotherapy	autologous dendritic cells electroporated with mRNA	Radboud University Medical Center	IV	I/II	NCT00929019	2009
Advanced pancreatic carcinoma	T cells	Immunotherapy	CD276 CAR‐T cells	Shenzhen University General Hospital	IV	I/II	NCT05143151	2021
Hepatocellular carcinoma	T cells	Immunotherapy	LioCyx‐M	Lion TCR Pte. Ltd.	IV	I	NCT04745403	2021
Refractory malignant solid neoplasm	T cells	Immunotherapy	Anti‐MESO CAR T cells	Ruijin Hospital	IV	I	NCT04981691	2021
Solid tumors	NK cells	Immunotherapy	CAR‐NK cells targeting NKG2D ligands	The Third Affiliated Hospital of Guangzhou Medical University	Electroporation	I	NCT03415100	2018
Adrenal cortical carcinoma/medullary thyroid cancer/thymic neuroendocrine carcinoma/pancreatic neuroendocrine tumor	N/A	Immunotherapy	mRNA‐0523‐L001	Shanghai Jiao Tong University School of Medicine	IM	N/A	NCT06141369	2023
Advanced esophageal squamous carcinoma/gastric adenocarcinoma/pancreatic adenocarcinoma/colorectal adenocarcinoma	N/A	Immunotherapy	Personalized mRNA Tumor Vaccine	Changhai Hospital	SC	N/A	NCT03468244	2018
Advanced solid tumor	N/A	Immunotherapy	mRNA‐0217/S001	Ruijin Hospital	N/A	I	NCT05916248	2023
Digestive system neoplasms	N/A	Immunotherapy	iNeo‐Vac‐R01	Sir Run Run Shaw Hospital	SC	I	NCT06026800	2023
Malignant melanoma	N/A	Immunotherapy	mRNA coding for melanoma associated antigens	University Hospital Tuebingen	SC	I/II	NCT00204516	2005
Malignant mesothelioma/colorectal cancer/bile duct cancer/rectal cancer/ovary cancer/pancreatic cancer/breast cancer female	N/A	Immunotherapy	UCMYM802	UTC Therapeutics Inc.	IV	I	NCT06256055	2024
Solid tumor	N/A	Immunotherapy	Neoantigen mRNA Vaccine	Second Affiliated Hospital of Guangzhou Medical University	Local injection	I	NCT06195384	2023
Liver cancer	N/A	Immunotherapy	Neoantigen mRNA Personalized Cancer vaccine	Shanghai Zhongshan Hospital	SC	N/A	NCT05761717	2023
**Metabolic diseases**								
Glycogen storage disease	LNPs	Protein‐replacement therapy	mRNA‐3745	ModernaTX, Inc.	IV	I/II	NCT05095727	2021
Acne	N/A	Immunotherapy	Acne mRNA vaccine	Sanofi Pasteur, a Sanofi Company	IM	I/II	NCT06316297	2024
**Virus**								
Cytomegalovirus infection	LNPs	Immunotherapy	mRNA‐1647	ModernaTX, Inc.	IM	III	NCT05085366	2021
Virus diseases	LNPs	Immunotherapy	CoV2 SAM (LNP)	GlaxoSmithKline	IM	I	NCT04758962	2021
**Cardiovascular diseases**								
Healthy volunteers	LNPs	Immunotherapy	mRNA‐6231	ModernaTX, Inc.	SC	I	NCT04916431	2024
Chronic heart failure	LNPs	Immunotherapy	mRNA‐0184	ModernaTX, Inc.	IV	I	NCT05659264	2022
**Rare genetic diseases**								
Methylmalonic acidemia	LNPs	Protein‐replacement therapy	mRNA‐3705	ModernaTX, Inc.	IV	I/II	NCT04899310	2021
Phenylketonuria	LNPs	Protein‐replacement therapy	mRNA‐3210	ModernaTX, Inc.	IV	I/II	NCT06147856	2023
Propionic acidemia	LNPs	Protein‐replacement therapy	mRNA‐3927	ModernaTX, Inc.	IV	I/II	NCT04159103	2019
Transthyretin amyloidosis	LNPs	Gene editing therapy	NTLA‐2001	Intellia Therapeutics	IV	I	NCT04601051	2020
Transthyretin amyloidosis	LNPs	Gene editing therapy	NTLA‐2001	Intellia Therapeutics	IV	III	NCT06128629	2023
Hereditary angioedema	LNPs	Gene editing therapy	NTLA‐2002	Intellia Therapeutics	IV	I/II	NCT05120830	2021
Hereditary angioedema	LNPs	Gene editing therapy	NTLA‐2002	Intellia Therapeutics	IV	III	NCT06634420	2024
Familial hypercholesterolemia	Exosomes	Protein‐replacement therapy	Low Density Lipoprotein Receptor mRNA Exosomes	Tang‐Du Hospital	IV / IP	I	NCT05043181	2021

Abbreviations: IV, intravenous injection; IM, muscle injection; IP, intraperitoneal injection; SC, subcutaneous injection; ID, intradermal injection.

Data sources—ClinicalTrials. gov.

Clinical studies have shown that Zika virus infection may be preventable with mRNA‐based vaccines. Moderna has developed an mRNA vaccine (mRNA‐1893) that encodes the structural proteins of the Zika virus. It was able to generate neutralizing antibodies in its initial phase I study (NCT04064905), while phase II launched in 2021 (NCT04917861) with over 800 people. With the success of COVID‐19 mRNA vaccines, HIV will be the next obstacle to overcome. Up to now, several HIV mRNA vaccines have been constructed and are now in clinical trials (NCT06375512, NCT05001373, NCT00833781). These vaccines are intended to express the antigens simulating the binding site of HIV, which can activate specific progenitor B cells to trigger broad‐spectrum neutralizing antibodies against HIV in the body. mRNA vaccines can activate specific immune responses by encoding an antigen protein. Developing mRNA vaccines against infectious diseases requires only changing the mRNA sequence encoded relevant antigens. It makes mRNA vaccines a great advantage in the influenza vaccines. The efficacy, immunogenicity, and safety of influenza‐related mRNA vaccine candidates are still investigated in multiple clinical trials listed in Table 1.

The antitumor drugs based on immunotherapy are currently the most developed pipeline of mRNA therapeutics. Tumor antigen is the key to the immunogenicity of the mRNA vaccine. Moderna has developed a personalized tumor vaccine (mRNA‐4157) encoded up to 34 kinds of neoantigens, which targeted patient‐specific mutations. Phase II results (NCT03897881) of mRNA‐4157 showed a 44% reduction in the risk of melanoma recurrence or death after treatment, while mRNA‐4157 has been in phase III. In addition, the progression of some tumors is highly correlated with viral infection, such as Epstein–Barr virus (EBV)‐positive nasopharyngeal carcinoma and viral hepatitis type B (HBV)‐positive liver cancer. Our group has constructed two mRNA vaccines against EBV‐positive advanced malignant tumors and HBV‐positive liver cancer, which have been in phase I (NCT05714748, NCT05738447).

The development of gene‐editing therapy provides promise for curing rare genetic diseases. mRNA drugs can quickly express encoded gene editors in target cells. Compared with the delivery of protein editors, mRNA therapeutics will achieve more long‐term effects. Intellia Therapeutics has developed two types of mRNA drugs based on gene‐editing therapy: transthyretin amyloidosis (ATTR, NTLA‐2001) and hereditary angioedema (NTLA‐2002). This therapy involves the administration of LNP‐encapsulated mRNA encoding cas9 protein and gRNA targeting the disease gene. The two mRNA drugs have both achieved good therapeutic effects in clinical phase I/II (NCT04601051, NCT06128629) and are being developed in clinical phase III (NCT05120830, NCT06634420).

At present, mRNA‐based therapy has also been used to treat metabolic diseases (NCT05095727, NCT06316297) and cardiovascular diseases (NCT04916431, NCT05659264), but they are in the early clinical stage. We look forward to the results of these clinical trials.

Although so many mRNA drugs have entered the clinic, there are still only two vaccines for infectious diseases on the market. Insufficient delivery efficiency and bio‐safety are the important factors for this situation, which can be greatly improved through targeted delivery systems. Clinical trials have found that LNP‐based mRNA vaccines will trigger unwanted inflammatory responses. On the other hand, mRNA drugs have been on the market for only 2 years, and whether repeated dosing causes lipid accumulation in target or nontarget tissues remains to be explored. Therefore, the development of effective humanized model animals will improve the preclinical evaluation of mRNA drugs and their delivery systems, which can accelerate the clinical transformation of mRNA drugs. In addition, the large‐scale production, quality control, effectiveness evaluation, pharmacokinetics, and safety studies of mRNA drugs are also crucial for their clinical transformations.

## 4 THE ADVANCES IN TARGETED DELIVERY OF mRNA

**FIGURE 5:**
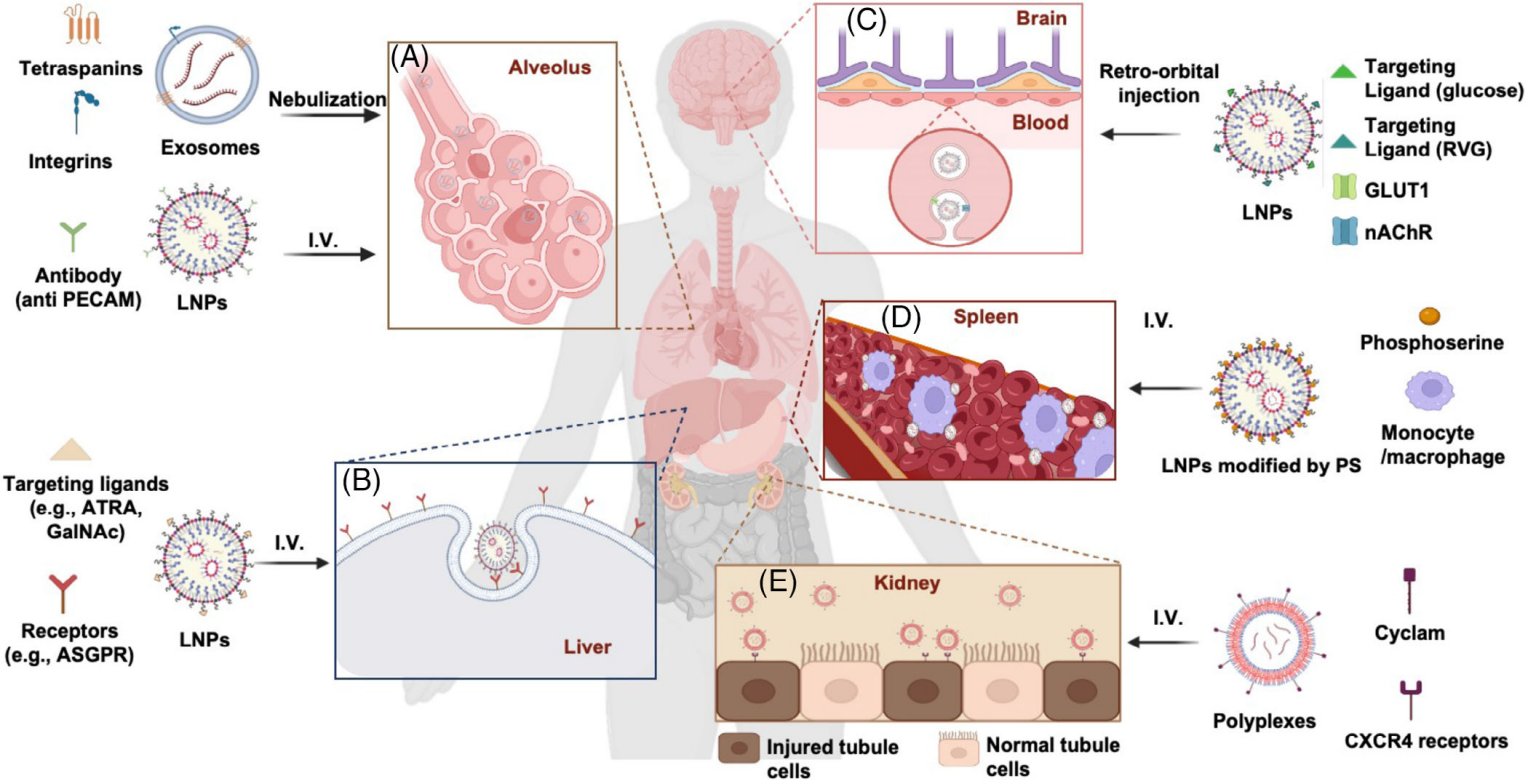
The various applications of targeted mRNA delivery systems. (A) Lung‐targeted applications. Exosomes are decorated with various integrins and tetraspanins via the nebulization route. LNPs bind specific antibodies (e.g., anti PECAM) via the intravenous route. (B) Liver‐targeted applications. LNPs bind the targeting ligand (e.g., ATRA, GalNAc) via the intravenous route. (C) Brain‐targeted applications. LNPs bind the targeting ligand (e.g., glucose, RVG) via retro‐orbital injection; binding of glucose to GLUT1 and RVG to nAChR allows LNPs to be endocytosed by endothelial cells. (D) Spleen‐targeted applications. MC3‐based LNPs modified by PS are delivered to the spleen by binding to macrophages via the intravenous route. (E) Kidney‐targeted applications. Polyplexes decorated with cyclam bind to CRCX4 receptors for delivery to injured tubule cells via the intravenous route. Biorender was used for this figure.

**FIGURE 6:**
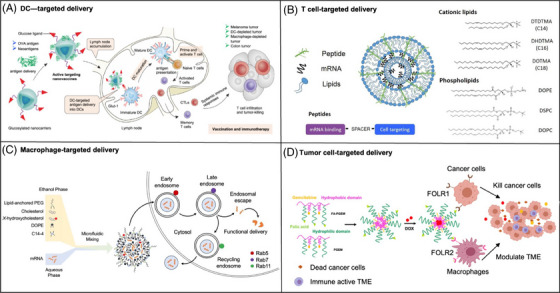
The applications of cellular targeted delivery. (A) Dendritic cells. Glucosylated nanovaccines targeting Glut‐1 on DCs deliver both conventional antigens and tumor‐specific neoantigens, triggering DCs maturation and robust adaptive immune responses. (B) T cells. The synthesis of LNPs engineered to transport mRNA to T cells was achieved by substituting cholesterol with hydroxycholesterol within their structure and design.^228^ (C) Macrophages. The dual targeting of both tumor cells and tumor‐associated macrophages in breast cancer therapy developed by utilizing folate receptors highly expressed on these cells.^234^ (D) Tumor cells. Lipid–peptide nanocomplexes to deliver mRNA to the murine B16‐F10 melanoma tumor.^235^

mRNA therapeutics have found extensive applications in protein replacement therapy and gene editing therapy. In addition, according to the indications and mechanisms of mRNA drugs, it is crucial to select the appropriate delivery vector, as it significantly impacts the therapeutic effects. For example, the application of immune organs (e.g., spleen, lymph nodes) targeted mRNA delivery is an efficient approach to improve the efficacy of mRNA vaccines against infectious diseases and tumors.^129^ Over the past few decades, various development directions of targeted carriers have been proposed, including optimizing ionizable lipids, replacing constituent lipids, and adding targeting ligands. This section summarizes the recent advances in tissues (Figure 5) and cells (Figure 6) targeted delivery systems, which were also listed in Table 2. It should be noted that various administration routes directly result in differences in targeting profiles. This section primarily focuses on improving the targeting ability of mRNA delivery systems administered intravenously.

**TABLE 2:** List of the tissue/cell‐targeted delivery systems.

Targeted tissues/cells	Delivery systems	References
Lung	Polyesters modified with different alkyl chain lengths and molar ratios (PE4K‐A17‐0.2C8)	^130^
	Nanoparticle platform comprising ionizable N‐methyldiethanolamine and kinds of hydrophobic alkyl diols	^131^
	Five‐element nanoparticles incorporating PBAEs and DOTAP	^132^
	Polyaspartamide derivative with a cyclohexyl ethyl group (PAsp(DET/CHE))	^133^
	Poly‐β‐amino‐thio‐ester (PBATE) (P76)	^134^
	DOTMA/CHOL	^135^
	LNPs with 55% DODAP	^136^
	IAJDs with modification	^140^, ^141^
	NPs bound to RBCs	^132^, ^133^, ^134^, ^135^, ^136^, ^137^, ^138^, ^139^, ^140^, ^141^, ^142^, ^143^, ^144^, ^145^
	LNPs with SATA‐maleimide conjugation	^146^
	A nanoparticle with a high content of PEG‐lipid and cationic helper lipid (NLD1)	^148^
	LNPs with increasing PEG concentration and a cholesterol analog, β‐sitosterol	^149^
	A PEGylated shielding shell and a bifunctional peptide‐modified corona, containing KGF	^150^
Liver	LNPs (306Oi10)	^159^
	LNPs (PPZ‐A10‐based formulations)	^160^
	LNPs composed of disulfide bond‐containing hydrophobic tails	^161^, ^162^
	LNPs with ASGPR mAb	^167^
	LNPs doped with 3% mannose PEG‐lipid	^169^, ^170^
	A glycolipid‐like polymer named galactosylated chitosan oligosaccharide‐SS‐octadecylamine (Gal‐CSSO)	^171^
Spleen	LNPs with a DODAP/Chol ratio of 48.5/0 mol% and a DOPE/DODAP ratio of 60/28.5 mol%	^137^
	Medium‐sized (∼200–320 nm) complex (RNA‐LPX) with a lowered charge ratio (≤1.7:2)	^173^
	MC3‐based LNPs with PS	^180^
	LNPs bind to CD4 antibodies	^181^
	LNPs (substituting DOPE with DSPC)	^183^
	LNPs with low PEG lipid, high ionizable lipid (Coatsome SS‐EC), and low DOPE content	^184^
	Zwitterionic phospholipidated polymers (ZPPs)	^186^
	LNPs (113‐O12B)	^187^
	Charge‐altering releasable transporters (CARTs)	^189^, ^190^
Kidney	3‐µm sized fluorescent capsules composed of poly‐L‐arginine and dextran sulfate	^193^
	Nanoparticles with sizes of 30–80 nm modified by kidney‐targeted peptides (CSAVPLC)	^196^
	Zwitterionic peptide ligand (KKEEE)3K	^197^, ^198^, ^199^, ^200^
Eye	Ionizable lipids with unsaturated hydrocarbon tails	^203^
	A pH‐responsive hybrid nanoparticle (SMOF NP)	^168^, ^204^
Brain	SNCs with glucose and rabies virus glycoprotein peptide	^208^
	Particles modified with interleukin receptor, NRP‐1 receptor, brain‐targeted peptides TAT48‐61, Angiopep‐2, and DA–PEI carriers	^209^, ^210^, ^211^, ^212^
Gastrointestinal tract	Branched hybrid poly(β‐amino ester) nanoparticles	^213^
Dendritic cells	Particles modified with C‐type lectin receptors (e.g., DCs‐SIGN, mannose receptor), DEC205, and Clec9A	^214^, ^215^, ^216^
	Engineered nanoparticles	^217^, ^220^, ^221^
T cells	C14‐4 LNPs	^226^
	Lipid nanoparticle B10	^227^
	LNPs with 7α‐hydroxycholesterol.	^228^
	Anti‐CD3‐targeted lipid nanoparticles (aCD3‐LNPs)	^229^
	Ag‐presenting nanoparticles (APNs)	^230^
Macrophages	Nanoparticles comprised of a poly(lactide‐co‐glycolide) core and an acid‐sensitive sheddable polyethylene glycol (PEG) moiety	^232^
	GM3‐NPs	^233^
	The ultra‐small‐sized gemcitabine‐based NPs modified by folic acid	^234^
Tumor cells	LNPs containing a cationic lipid with a fourteen‐carbon tail and a modified peptide component (K16GACYGLPHKFCG)	^235^
	EVs bound with anti‐HER2 scFv	^236^, ^237^
	RBC‐derived EVs	^238^

### 4.1 Tissue‐targeted delivery

#### 4.1.1 Lung‐targeted delivery

Recent advancements in mRNA delivery show promise for treating lung‐related diseases through improved targeting, but safe and efficient lung delivery methods remain challenging. Strategies like using LNPs and EVs have been explored for lung‐targeted mRNA delivery, with varying levels of success. However, concerns regarding toxicity, inflammation, and uptake by lung macrophages persist and need to be addressed. Despite these hurdles, the potential applications for lung‐targeted mRNA delivery are substantial, encompassing conditions such as pulmonary fibrosis, cystic fibrosis (CF), and lung cancer treatment.

Different structures of the carriers are optimized differently to obtain efficient lung aggregation. Yu et al.^130^ established a functional polyester library, focusing on hydrophobic optimization. Among various polyesters modified with different alkyl chain lengths and molar ratios, PE4K‐A17‐0.2C8 emerged as the optimal mRNA carrier for lung delivery. In addition, the mRNA delivery nanoparticle platform comprising ionizable N‐methyldiethanolamine (MDET) and kinds of hydrophobic alkyl diols had the potential to selectively deliver mRNA to lung alveolar macrophages and dendritic cells (DCs).^131^ Recently, five‐element nanoparticles incorporating PBAEs and DOTAP were designed, which showed that PBAEs with E1 end‐caps, higher degrees of polymerization, and longer alkyl side chains had efficient lung‐targeted ability.^132^ Besides, there are a lot of vector optimization for effective lung‐targeted transfection, such as the polyaspartamide derivative with a cyclohexyl ethyl group (PAsp(DET/CHE)), and the poly‐β‐amino‐thio‐ester (PBATE) named P76.^133^, ^134^ According to the method of using CRE reporter mice, Rosigkeit et al.^135^ showed that the selected DOTMA/CHOL Cre mRNA preparations were preferentially delivered to pulmonary endothelial cells and tissue‐resident alveolar macrophages rather than leukocytes.

Selective organ targeting (SORT) design and its content adjustment can change the biological distribution of LNPs to different tissues. For instance, 5A2‐SC8 SORT LNPs were originally formulated with 20% DODAP, resulting in effective PTEN editing in the liver, a widely expressed tumor suppressor. Interestingly, when the DODAP content was increased to 55%, exclusive PTEN editing occurred in the lungs.^136^ In a separate study, higher gene expression was observed in the spleen with a DODAP/Chol ratio of 48.5/0 mol% and a DOPE/DODAP ratio of 60/28.5 mol%.^137^ Researchers developed iPhos lipids with a single tertiary amine, a phosphate group, and three alkyl tails to enhance protein expression and achieve organ‐selective mRNA delivery as SORTs. They found that the amine chain length influenced delivery efficacy, while the alkyl chain length near the phosphate group determined organ selectivity. The iPhos could selectively deliver mRNA to the spleen, liver, and lungs by interacting with various accessory lipids.^138^ In addition, Mitchell et al.^139^ developed a barcoded high‐throughput screening system to identify the lung‐targeting efficacy of novel ionizable lipids. They first combinatorically synthesized 180 ionizable lipids, and then used barcoding technology to quantify how these nanoparticles deliver DNA barcodes in vivo. The top‐performing nanoparticle formulation delivering mRNA‐encoded genetic editors exhibited great therapeutic effects for antiangiogenic cancer therapy in the lung tumor model.

Modification of hydrophilic and hydrophobic sequences can affect the propensity of carriers to aggregate in the lungs. The original architecture of one‐component ionizable amphiphilic Janus dendrimers (IAJDs) were inspired by the structure of amphiphilic Janus dendrimers, Janus glycodendrimers (JGDs), and sequence‐defined JGDs self‐assembling. Protonated ionizable amines are the key to alter delivery from the lung to the spleen or liver. Substitution of the interconnecting ester with the amides showed more selective delivery to the lung, while the pairs of odd and even alkyl groups in the hydrophobic dendron tended to target the liver.^140^ IAJDs modified by single–single (SS, single hydrophilic dendron connected to single hydrophobic dendron), a twin–twin (two hydrophilic dendrons connected to two hydrophobic dendrons), and twin‐mixed (two different hydrophilic dendrons connected to two hydrophobic dendrons), have been found that there were some carriers efficaciously for targeted delivery to the lung.^141^

As for the lung‐targeted delivery, addition of specific ligands is effective. Noncovalent binding with RBCs is a strategy to extend NP circulation time and achieve selective absorption by endothelium‐rich organs.^142^ Researchers developed the NPs containing positively charged chitosan or PLGA tightly bound to RBCs, which were mainly absorbed by the lung rather than the liver and spleen.^143^, ^144^ The design of β‐cyclodextrin modified RBCs and ferrocene‐modified NPs co‐incubated played a more effective role in solid combination.^145^ While not applied to mRNA drug therapy yet, these RBC‐based methods can be innovative in mRNA delivery. Moreover, in pursuit of precise lung targeting, monoclonal antibodies (mAbs) designed to specifically bind to PECAM‐1 were covalently attached to LNPs using SATA‐maleimide conjugation chemistry. The resultant antibody‐targeted LNP‐mRNAs exhibit an independent route of uptake, distinct from the apo‐E mediated pathway that directs them to the liver.^146^

The inhaled vaccine can stimulate not only humoral and cellular immunity but also efficacious respiratory mucosal immunity with less dose. Given the difficulties in the treatment of pulmonary diseases such as COVID‐19, acute lung injury, and CF, the inhaled vaccine has become a hot spot of current research. Physiologic barriers to the inhalation pathway in the lungs, such as the mucus layer, respiratory epidermal cells, and macrophages, affect the deposition and penetration of mRNA delivery vectors. Furthermore, owing to shear stress during aerosolization that caused LNPs disintegration, it requires improvements in delivery carriers to achieve effective and stable delivery of aerosolized therapeutic mRNA to the lungs. The stable hyperbranched cationic polymer class of PBAEs has been reported to promote effective nebulized mRNA delivery to lung epithelium. But after a single dose, toxicity characterized by weight loss may be observed.^147^ A nanoparticle called nebulized lung delivery 1, designed with a high content of PEG‐lipid and cationic helper lipid, has been developed by researchers to deliver mRNA encoding a widely neutralizing antibody to the lung through nebulization.^148^ Another group has been working on delivering mRNA encoding therapeutic proteins to the lung through inhalation. In their recent study, they used LNPs with increasing PEG concentration and a cholesterol analog, β‐sitosterol, to achieve uniform particle distribution, polyhedral morphology, and rapid mucosal diffusion.^149^ Additionally, a different inhaled mRNA formulation with a PEGylated shielding shell and a bifunctional peptide‐modified corona, containing keratinocyte growth factor, was created for lung‐targeted delivery to help re‐epithelialize disrupted alveolar epithelium, potentially aiding in the repair of fibrotic foci in idiopathic pulmonary fibrosis.^150^ Furthermore, it was found that using lung‐derived exosomes for mRNA loading in inhalation therapy increased uptake by lung parenchyma cells compared with liposomes.^151^

These studies lay the groundwork for advancing targeted mRNA delivery systems in pulmonary disease treatment. Future research should focus on characterizing diverse mRNA delivery methods tailored to specific lung cell types. This is essential to reduce lung damage, minimize drug toxicity, and prevent uptake by pulmonary macrophages, ultimately prolonging therapeutic efficacy in the lungs. Pulmonary delivery efficiency can be enhanced through route alterations like inhaled vaccines. Simultaneously, it is essential to research strategies for reducing the proinflammatory effects linked to LNPs. Addressing these challenges will lead to the development of safer and more effective targeted mRNA delivery systems for pulmonary therapy. Furthermore, differences in lung anatomy between mice and humans may impact the performance of lung‐targeted delivery carriers in clinical trials, underscoring the need for additional research using animal models to deepen our understanding of the underlying mechanisms of targeted mRNA delivery to the lungs.

#### 4.1.2 Liver‐targeted delivery

The liver constitutes a vital organ that assumes a pivotal role in various physiological processes encompassing metabolism, detoxification, and immune modulation. However, liver diseases such as hepatitis, cirrhosis, and liver cancer are prevalent and can be life‐threatening. LNPs have been shown to selectively accumulate in the liver due to their uptake and clearance by liver macrophages, the largest population of phagocytes in the body. Moreover, the interplay between LNPs and plasma proteins substantially influences their systemic clearance dynamics post‐administration. Therefore, liver‐targeted delivery using LNPs represents a promising avenue for the treatment of liver‐related diseases, as it can minimize the occurrence of side effects and improve the efficacy of therapeutic agents.

In the past few years, LNPs always selectively accumulate in the liver, which correlates with nanoparticle uptake and clearance by liver macrophages, the largest population of phagocytes in the body.^152^ In addition, the interaction of LNPs with plasma proteins plays an important role in the clearance process of LNPs after entering the body.^153^ Most of the studies on carriers selectively delivered to the liver are conducted from ApoE, which promotes receptor‐mediated cellular entry.^99^ A recent study indicated that the binding of ApoE can induce the rearrangement of the shell and core lipids of LNPs containing mRNA.^154^ According to Zheng et al.,^155^ the ability to attain liver targeting can be accomplished by systematically refining the nanoparticle vector formulation using the central composite design approach.

The human liver is primarily composed of parenchymal cells and nonparenchymal cells. Focusing on these specific cell types provides a precise approach to treating disease. Liver parenchymal cells are commonly implicated in hepatitis especially the HBV and other related diseases, whereas nonparenchymal cells, such as liver sinusoidal endothelial cells (LSECs) and hepatic stellate cells,^156^ are closely associated with chronic liver conditions like liver fibrosis and cirrhosis.^157^, ^158^ The LNPs such as the 306Oi10 LNPs and PPZ‐A10‐based formulations, have been designed for simultaneous transfection of liver cells, including hepatocytes, Kupffer cells, and endothelial cells.^159^ The ionizable lipids were synthesized with a piperazine core and two tertiary amines. During screening, researchers discovered PPZ‐A10, a lipid with shorter C10 carbon chains. They incorporated PPZ‐A10, along with cholesterol, C18PEG2K, and DOPE, to create LNPs for efficient mRNA delivery to Kupffer cells.^160^ Furthermore, a range of vector optimized strategies have been developed with the specific aim of improving the targeted delivery to liver parenchymal cells. A delivery vector designed by Finn et al.^161^ achieved significant hepatic editing, with around 70% effectiveness at the mouse thyroxine transfer protein gene locus. This editing reached most hepatocytes in the mouse liver, including GS+ pericentral cells.^161^ In another example, the reducible LNPs composed of disulfide bond‐containing hydrophobic tails have been screened for delivering Cas9 mRNA and single guide RNA (sgRNA) to hepatocytes to promote proprotein convertase subtilisin/Kexin type 9 (PCSK9) knockdown.^162^, ^163^ There was a design of LNPs targeting the hepatic reticuloendothelial system (RES) that enhanced mRNA expression in the liver, especially in RES cell types.^164^ Such targeting approaches hold potential for ameliorating liver‐related disorders by modulating specific cellular responses, thereby minimizing off‐target effects and fostering therapeutic efficacy.

Using ligands to actively target delivery vectors to the liver and other organs of interest is a potential research area. This approach holds the potential to mitigate the undesirable delivery of LNPs to extrahepatic organs, thereby minimizing the occurrence of side effects. The asialoglycoprotein receptor (ASGPR) is overexpressed on the surface of hepatoma cells. It has been suggested that ASGPR‐mediated endocytosis is an effective delivery strategy. The incorporation of the galactose group has been well‐documented to selectively bind to ASGPR into mRNA carriers.^165^, ^166^, ^167^, ^168^ Another idea is to conjugate the LNPs with ASGPR mAb, which may be an attempt.^167^ In a study by Kim et al.,^169^ it was found that achieving LSEC‐specific RNA delivery, could not succeed by controlling the size and PEG‐lipid content of LNPs. Due to the specific expression of mannose receptors on human and mouse LSECs, they prepared LNPs doped with 0.5–4.5% mannose PEG‐lipid, and found that incorporation of mannose to LNPs with high PEG‐lipid content (3%) has the best LSECs selective delivery effect.^169^, ^170^ Miao et al.^171^ have proposed a glycolipid‐like polymer named galactosylated chitosan oligosaccharide‐SS‐octadecylamine (Gal‐CSSO). The galactosyl residues modified on it can selectively target HCs, the chitosan oligosaccharide is conducive to the endosomal escape, and the structure of –SS– can help the HC microenvironment respond to facilitate drug release.

Many carriers with excellent liver selective delivery have been approved for drug delivery or have started clinical trials. Patisiran (ONPATTRO)™, the first siRNA drug in the world. It is encapsulated in LNPs and delivered to hepatocytes, which specifically inhibits the hepatic synthesis of transthyretin.^172^ A study of investigational in vivo CRISPR/Cas9 genome editing candidate, NTLA‐2001 (NCT04601051), is in phase I clinical trial, which is being developed as a single‐dose treatment for transthyretin (ATTR) amyloidosis by using LNPs for rapid distribution to the liver through the hepatic artery.^173^

#### 4.1.3 Spleen/lymph‐targeted delivery

Given the abundance of immune cells within the spleen and lymph nodes, which are important sites for producing antibodies and effector cells, investigating spleen and lymphoid organs specific targeting emerges as a promising strategy for the development of the next generation of mRNA vaccines. Moreover, macrophages are considered as the main cell population exposed to nanoparticles and induced internalization, which initiate these processes generally in the liver, spleen, and kidney. Current clinical mRNA vaccines can cause side effects in the liver, such as reversible liver injury and T‐cell dominant immune‐mediated hepatitis. Thus, by targeting the spleen and lymphoid organs, vaccine efficacy may be enhanced while minimizing adverse effects.

LNPs with adjusted charge, size optimization, and specific compositions allow precise DCs targeting within lymphatic compartments for potential antitumor responses. A kind of medium‐sized (∼200–320 nm) complex (RNA‐LPX) with a further lowered charge ratio (≤1.7:2) was designed to be primarily expressed in the spleen.^174^ Likewise, Sasaki et al.^175^ screened a wide range of particle sizes and indicated that a size range from 200 to 500 nm is appropriate for targeting splenic DCs. The incorporation of RAL1, RAL2, and TLR7/8 agonist resiquimod (R848) derived amino lipids successfully delivered CD40 mRNA to DCs for antitumor.^176^ Focus on optimizing phospholipid chemistry, the addition of phosphoethanolamine head group presumably increases endosomal escape.^175^ It has been proved that zwitterionic phospholipids mainly aided liver delivery, while negatively charged phospholipids provided the tropism of the LNPs to the spleen.^177^ Since phosphatidylserine (PS) is a signaling molecule that can be used to enhance cellular uptake, a study of mRNA delivery vector tried to replace DOPE with PS, then the positive charge was reduced by half, even changing the protein expression ratio of the liver to the spleen from 8:1 to 1:3.^178^, ^179^ Similarly, when PS was incorporated directly into MC3‐based LNPs, effective protein expression was detected in both lymph nodes and spleen after intravenous administration.^180^ Conjugating the CD4 antibody to LNPs has proven promising, as it significantly increased radiolabeled mRNA accumulation in the spleen and lymph nodes, resulting in approximately a 30‐fold higher signal for reporter mRNA.^181^ As for the design of ionizable lipids with spleen‐targeted ability, Chen et al.^182^ found that ionizable lipids equipped with branched and biodegradable tails could enhance the selective delivery of mRNA to spleen. The lipid A28‐C6B2 was identified with high spleen‐specific mRNA expression via intravenous injection. What is more, A28‐C6B2 could efficiently target mRNA delivery to antigen‐presenting cells, which might have great potential utility in immunotherapy. This study offered a novel insight into how the chemical structure of ionizable lipids influenced the targeting capabilities of LNPs.

There are various techniques and technologies that can optimize the molar ratio of lipids and enhance lymphocyte transfection and T cell response. Researchers have used computer‐aided methods, DNA barcoding, and quality‐by‐design approach based on a statistical design‐of‐experiment coupled with Bayesian regression modeling to optimize the molar ratio of lipids in LNPs.^175^, ^183^, ^184^ Through these approaches, substituting DOPE with DSPC as a helper phospholipid significantly increased mRNA‐LNPs accumulation in the spleen.^185^ LNPs with low PEG lipid, high ionizable lipid (Coatsome SS‐EC), and low DOPE content yielded the most enhanced T cell response, with spleen DC and macrophages having the highest percentage of transfected cells.^184^

Additionally, various polymeric platforms with different formulations have been used for spleen and lymph node delivery. Liu et al.^186^ synthesized zwitterionic phospholipidated polymers via cationic polymer postmodification as mRNA delivery carries, which mediated increased protein expression in vitro and enhanced mRNA targeted delivery to the spleen and lymph nodes following intravenous administration. A recent study proposed LNPs named 113‐O12B for cancer immunotherapy, which collected molecules from the blood stream on its surface, and these selected molecules bind to specific receptors in target organs. 113‐O12B was identified to competently deliver both a full‐length protein and a short‐peptide–based, antigens‐encoded mRNA to lymph nodes, eliciting robust CD8 T cell responses.^187^ Compared with liposome–mRNA complexes (LRCs) and LRCs assembled with hyaluronic acid (HLRCs), it was found that using HLRCs led to a faster decrease of mRNA accumulation ratio in the liver and spleen.^188^ Charge‐altering releasable transporters delivery system is an optional polymeric platform to enhance lymphocyte transfection in primary T cells as delivery carries.^189^, ^190^ The small‐molecule drug fingolimod (FTY720), which can bind to the sphingosine‐1‐phosphate receptor 1 (S1P1) that is highly expressed on lymphocytes, led to the increment of mRNA delivery to marginal zone B cells and NK cells in the spleen.^191^

#### 4.1.4 Kidney‐targeted delivery

Kidney diseases encompass a wide spectrum of conditions, ranging from chronic kidney diseases to acute conditions like nephritis and renal cancer. The human kidney consists of numerous nephrons containing a renal corpuscle. Its associated tubules play a critical role in filtering blood and removing waste from the human body.^192^ The challenge of targeted drug delivery to the kidney is compounded by the need to navigate through various barriers, including the glomerular filtration barrier, tubular reabsorption, and potential off‐target effects on other organs. Strategies such as nanoparticle‐based drug carriers, ligand–receptor interactions, and innovative renal‐specific drug formulations are being explored to overcome these hurdles. These approaches aim to enhance drug accumulation at the site of action, improve therapeutic efficacy, and mitigate potential adverse effects on nontarget organs.

Recent studies have demonstrated that 3‐µm sized fluorescent capsules composed of poly‐l‐arginine and dextran sulfate can effectively target the kidney via a mice renal artery.^193^ The cationic polymer‐based carrier, polyplex nano‐micelles, was employed to deliver mRNA to the kidney via a renal pelvis injection.^194^

Whereas nanoparticles are still the main object of targeted delivery research. Previous studies have indicated that nanoparticles smaller than 10 nm can pass through the glomerular filtration membrane, yet they are mainly captured by the liver and spleen when larger than 100 nm.^195^ It has been suggested that nanoparticles with sizes of 30 ∼ 80 nm were effectively delivered to the kidney when modified by kidney‐targeted peptides with the unique sequence of CSAVPLC, which has been proven to be effective in recognizing and promoting endocytosis of renal cells.^196^ Except for chemical modification such as folic acid, PEG, and chitosan, the targeting peptides are also employed to attach nanoparticles, such as the zwitterionic peptide ligand (KKEEE)_3_K which has been proved to bind to megali that are broadly expressed in proximal tubule cells.^197^, ^198^, ^199^, ^200^ With the incorporation of (KKEEE)_3_K, a study developed the small, organic nanoparticles called peptide amphiphile micelles, successfully achieving kidney accumulation. Regrettably, it was observed that these micelles designed for kidney accumulation were also found to be delivered to the liver.^201^

Despite efforts by researchers to explore various compounds for kidney‐targeted drug delivery, there is a scarcity of studies focusing on the application of delivering mRNA drugs specifically to the kidney. This may be attributed to the fact that functional inhibition, achieved with siRNA and small molecule drugs, has been more extensively investigated as a viable treatment approach for kidney diseases. Presently, mRNA is primarily being studied in the context of protein replacement therapy. Conversely, the utilization of mRNA as a delivery system for CRISPR/Cas9 gene editing systems is still in its nascent stages, with ongoing studies aimed at exploring the efficacy of this approach.^202^ It is hoped that the optimization techniques for kidney‐targeted vectors may be applied to emerging mRNA therapy technologies.

#### 4.1.5 Others

*Eye*. Researchers have identified ionizable lipids with unsaturated hydrocarbon tails as effective for delivering mRNA to the retinal pigment epithelium (RPE) and Müller glia.^203^ Another method utilized a pH‐responsive hybrid nanoparticle (SMOF NP) composed of silica, zeolitic imidazole framework, and all‐trans‐retinoic acid (ATRA). This nanoparticle was designed to bind with interphotoreceptor retinoid‐binding protein and effectively deliver a CRISPR/Cas9 mRNA system to the RPE.^204^ They also optimized a kind of compound named SNP‐PEG‐ATRA modified by PEG for selective transport to the RPE.^168^ However, another study used ribonucleoproteins to deliver the complex composed of chemically modified sgRNA and SpCas9 protein to RPE, resulting in observed signs of toxicity.^205^

*Brain*. Research on mRNA delivery has also been gradually carried out in the treatment of drug delivery in the brain. However, for brain diseases such as aneurysmal subarachnoid hemorrhage, it is the primary difficulty to deliver drugs through the blood–brain barrier (BBB). Owing to the limited size and loading capacity, the application of EVs has been generally concentrated on the delivery of small RNA such as micro RNA and siRNA rather than mRNA, though the active loading of cargo molecules with mRNA length (>1.5  kb) is theoretically possible.^206^ Therefore, the main applications are still focused on nanoparticles. Dhaliwal et al. synthesized the cationic liposomes for mRNA encapsulation, which achieved the preferential distribution of the GFP signal intensity in the cortex and midbrain regions of the brain compared with the naked mRNA or other vehicle‐treated group within 24 h postadministration.^207^ In another study, modified tumor necrosis factor‐related apoptosis‐inducing ligand mRNA was intracranially delivered to the brain in a xenografted glioma mouse model and significantly inhibited tumor growth. Wang et al.^208^ have been committed to developing glutathione (GSH)‐responsive silica nanocapsules (SNCs). When conjugation of glucose and rabies virus glycoprotein peptide into SNCs, it bypassed the intact BBB and showed the effective brain delivery of the CRISPR/Cas9 genome. In addition to the above mentioned, there have been proposed other receptor‐modified brain‐targeted methods to solve the difficulty of BBB, such as interleukin receptor, NRP‐1 receptor, brain‐targeted peptides TAT48‐61, Angiopep‐2, and DA–PEI carriers.^209^, ^210^, ^211^, ^212^

*Gastrointestinal tract*. Patients tend to have better acceptance and adherence to oral medications compared with injectable drugs. To achieve this, researchers developed orally administered pills capable of delivering mRNA nanoparticle formulations with high transfection efficiencies to the gastrointestinal tract. This development followed the synthesis and screening of a library of branched hybrid PBAE mRNA nanoparticles.^213^

### 4.2 Cell‐targeted delivery

#### 4.2.1 Dendritic cells

DCs are ideal targets for mRNA delivery systems because of their capacity to process and present antigens to T cells, leading to the activation of CD4+ and CD8+ T cells, which are vital for robust immune responses. DCs reside in peripheral tissues and secondary lymphoid organs like lymph nodes, where antigen presentation occurs, making it critical for delivery systems to target these cells effectively.^214^, ^215^ Various strategies have been employed to improve DC‐targeted ability, including receptor‐mediated targeting using ligands or antibodies that bind specifically to receptors expressed on the surface of DCs, such as C‐type lectin receptors (e.g., DC‐SIGN, mannose receptor), DEC205, and Clec9A.^215^, ^216^, ^217^

The DC‐targeted mRNA nanocarriers have significantly improved uptake, processing, and presentation for the antigens. For instance, LNPs have been engineered to encapsulate mRNA while providing protection from enzymatic degradation, facilitating efficient delivery to DCs in vivo.^217^ Some systems employ mannose‐mimicking glycopolymers or glucose‐targeted nanovaccines, which exploit the glucose transporters and mannose receptors highly expressed on DCs to enhance specific uptake and antigen presentation.^216^, ^217^ These strategies have demonstrated enhanced DCs activation, antigen cross‐presentation via MHC class I and class II pathways, and potent T‐cell priming, leading to robust antitumor and antiviral responses.^217^, ^218^, ^219^

Moreover, novel platforms like in situ tumor vaccines have been developed, where engineered nanoparticles concentrate DCs at the injection site, thereby enhancing lymph node migration and boosting antigen‐specific immune responses.^217^, ^220^, ^221^ These approaches aim to improve the delivery of mRNA vaccines and modulate the tumor microenvironment, overcoming immune suppression and promoting sustained antitumor immunity.^221^, ^222^

#### 4.2.2 T cells

At present, gene therapy still has untapped potential to eradicate cancer. Cancer cells can escape from the attack of the immune system on account of some receptor's expression. T cells are the main force that damages tumor cells, becoming the research hotspot in cancer treatment such as chimeric antigen receptor T‐cell.^223^, ^224^, ^225^ After selecting the distinct ionizable lipids, the optimized C14‐4 LNPs were utilized to encapsulate CAR mRNA for the transfection of primary T cells to produce CAR T cells, and reduced cytotoxicity compared with electroporation.^226^ In one study, sequential libraries of different ionizable LNPs with varied excipient compositions were screened, leading to the identification of B10 as the best LNP for mRNA delivery to T cells. This LNP reduced cytotoxicity and enhanced its effectiveness in killing cancer cells.^227^ In another study, LNPs targeted CD5 to deliver modified mRNA, resulting in the generation of antifibrotic CAR T cells.^225^ Additionally, Patel et al.^228^ improved LNPs by replacing cholesterol with 7α‐hydroxycholesterol. This resulted in a significant 1.8‐fold and 2.0‐fold enhancement in mRNA delivery to primary human T cells ex vivo when substituting 25 and 50% of 7α‐hydroxycholesterol, respectively.

Besides, targeting T cells to deliver antigen‐coded mRNA can activate T cells and enhance the antitumor effect. Antibody–drug conjugate is a kind of promising targeting cancer therapy, it has been proved that optimized anti‐CD3‐targeted LNPs (aCD3‐LNPs) activated and consumed splenic and circulating T cells, as well as temporarily downregulation of CD3e ligand expression. It was an effective method that CD3 antibody was conjugated to the surface of LNPs, whereas it might lead to intricate immunological consequences.^229^ Hence, further research on potential immunological reactions and therapeutic mechanisms is necessary. The accuracy of T cell delivery can be extended to different peptide epitopes, so through the approach to deliver mRNA, a series of applications from in situ manufacturing of T cell therapy to genome editing and regulation can be realized. Su et al.^230^ synthesized Ag‐presenting nanoparticles by using UV light‐mediated ligand exchange for mRNA delivery to multiple influenza‐specific CD8+ T cells in the model of recombinant influenza A viral infection.

#### 4.2.3 Macrophages

The regulation of macrophage function can activate or silence inflammation‐related signal pathways. Passive targeting leverages nanoparticle properties and biological conditions, while active targeting uses ligands to bind specific macrophage receptors for precise mRNA delivery.^231^ Kraynak et al.^232^ designed the nanoparticle comprised of a poly(lactide‐co‐glycolide) core and an acid‐sensitive sheddable PEG moiety, coated with PS‐supplemented cell plasma membrane, which achieved the delivery to chronic inflammation and was preferentially taken up by macrophages. Moreover, there are still many vector designs targeting macrophages have been proposed, such as the Ganglioside monosialodihexosylganglioside (GM3)‐presenting lipid‐coated polymer nanoparticles targeting macrophages, and the ultra‐small‐sized gemcitabine‐based NPs modified by folic acid both targeting to tumor cells and tumor‐associated macrophages, while few applications of delivering mRNA.^233^, ^234^

#### 4.2.4 Tumor cells

Accurate delivery to tumor cells can enhance the efficacy of mRNA gene therapy. One method involved using LNPs containing a cationic lipid with a fourteen‐carbon tail and a modified peptide component (K16GACYGLPHKFCG), which successfully delivered genetic material to murine B16‐F10 melanoma tumors.^235^ In another approach, researchers harnessed extracellular EVs to transport HChrR6 encoded mRNA bound with anti‐HER2 scFv, leading to significant growth arrest in HER2‐positive human breast tumor xenografts in mice—an innovative use of EVs for delivering functional mRNA.^236^, ^237^ In lots of studies for drug delivery, EVs come from fibroblasts and DCs, but it is challenging to obtain these cells from subjects. Considering that EVs from whole plasma were safer, more abundant, and easier to obtain, a study used the RBC‐derived EVs to deliver the CRISPR–Cas9 genome editing system to leukemia and breast cancer cells in vitro and in vivo, resulting in no observable cytotoxicity, in contrast to the ∼20–30% increase in cell death caused by Lipofectamine™ 3000.^238^ Though the EVs derived from tumor cells have the advantage of preferential delivery to tumor cells due to their homotypic characteristics, attention should be paid to type selective uptake and the qualitative attributes of payload release.^239^ Additionally, targeting both tumor cells and the tumor microenvironment, including cancer‐associated fibroblasts, can enhance the effectiveness of mRNA‐based therapies by addressing multiple aspects of tumor progression and resistance.^240^

## 5 CHALLENGES AND PROSPECTS

mRNA therapeutics have shown great potential and been applied in clinics, and various types of delivery systems have been developed to protect and deliver mRNA in vivo. The expressing destination of the functional protein encoded by mRNA drugs depends on the indications and their therapeutic mechanism. For example, the antigens expressed in the spleen or lymph nodes specifically can enhance the immune therapeutics for the mRNA vaccines because the spleen or lymph nodes are rich in immune cells. Thus, developing mRNA medicines based on the targeted delivery systems can significantly enhance the transfection efficiency in specific sites and therapeutic effects. Meanwhile, the effective dose of the mRNA drugs will be reduced with the enhancement of the protein expression at the target sites, thereby improving their safety in vivo. In addition, this approach will reduce the off‐target effects and avoid the potential systemic toxicity. Although considerable progress has been made in developing targeted mRNA delivery systems, it is still far from clinical applications. There are still several challenges to be addressed.

When nanoparticles need to target specific organs or tissues, physiological obstacles must be crossed, especially for the brain, male reproductive system, and placenta, which are protected by special physiological barriers.^241^ Most targeting ligands were co‐conjugated with nanoparticles based on the PEG linkers, but high PEG densities are known to lead to unexpected immune responses in vivo.^242^, ^243^ Therefore, the balance between the efficiency and safety of targeted mRNA delivery vectors deserves further investigation.

Until now, we still lack a systematic understanding of how to overcome these obstacles via vector design. Nanoparticles will distribute systemically after intravenous injection, but the mRNA expression only occurs in a few tissues rather than all organs and tissues.^98^, ^105^ This phenomenon may suggest that the core of targeted delivery is controlling the cell type where cellular uptake and expression occur. Once nanoparticles enter the physiological environment, they are certain to interact with biomolecules in biological fluids, tissues, and cells. For example, nanoparticles will absorb humoral proteins in vivo to form protein corona on their surface, which affects their cellular uptake, biological distribution, and delivery efficiency. Thus, protein corona may provide a potential explanation for the targeting ability of nanoparticles and is worthy of deep exploration. It is reported that small changes in the structure of materials can indeed have important effects on the carrier, and the superposition of multiple molecules may amplify the differences in the overall properties. A better understanding of the interactions in several scenes is essential for the efficiency of the targeted delivery systems and the biosafety of nanomedicine.

On the other hand, the level of protein expression is the foundation of mRNA therapeutic effects. An available mRNA delivery system may be obtained from screening the hundreds of synthesized materials. However, the traditional strategies of synthesis and screening are usually time‐consuming, labor‐intensive, and susceptible to failure. Thus, the high‐throughput synthesis and screening (HTS) techniques for the mRNA drugs were applied based on the libraries containing ranging from 0.5 to 4 × 10^6^ compounds, which could greatly enhance the screening efficiency.^244^, ^245^ Chen et al.^246^ identified a newly structured lipid iso‐A11B5C1 through HTS that could centrally deliver mRNA to muscles and showed considerable transfection efficiency compared with commercial SM102 and MC3. Recently, artificial intelligence and machine learning approaches have been widely applied in designing and optimizing the mRNA sequence and delivery systems, which further improve the screening effectiveness of mRNA drugs in delivery (e.g., mRNA transfection efficiency, functional protein production), targeting effects (e.g., organs, tissues, cells, organelles), therapeutic effects (e.g., immune responses, antitumor or anti‐infection effects, reduction of disease symptoms), and safety (e.g., acute inflammatory response, acute hemolysis).^247^ Anderson et al.^248^ reported a novel approach to the high‐throughput screening of novel ILs for mRNA delivery via combinatorial chemistry and machine learning, and a novel lipid was finally designed by this strategy, which outperformed commercial ILs (MC3 and SM102) in transfecting muscle cells and immune cells in several tissues.^248^ These technologies provide a foundation for the improvement of targeted mRNA delivery and can be the popular direction for mRNA‐based medicines.

Finally, we hope this review can provide fundamental knowledge and rational guidance to accelerate the development of next‐generation targeted mRNA‐delivery systems. Meanwhile, it is anticipated that mRNA therapeutics will rapidly enter diverse clinical trials with the evolution of mRNA delivery techniques.

## AUTHOR CONTRIBUTIONS

*Conceptualization, data curation, writing—original draft*: Xi He. *Data curation, writing—original draft*: Guohong Li. *Data curation, writing—original draft*: Letao Huang. *Project administration, writing—review and editing*: Haixing Shi. *Validation, writing—review and editing*: Sha Zhong. *Validation, writing—review and editing*: Siyu Zhao. *Project administration, writing—review and editing*: Xiangyu Jiao. *Project administration, writing—review and editing*: Jinxiu Xin. *Writing—review and editing*: Xiaoling Yin. *Writing—review and editing*: Shengbin Liu. *Writing—review and editing*: Zhongshan He. *Writing—review and editing*: Mengran Guo. *Writing—review and editing*: Chunli Yang. *Project administration, writing—review and editing*: Zhaohui Jin. *Project administration, writing—review and editing*: Jun Guo. *Project administration, supervision, writing—review and editing*: Xiangrong Song. All authors have read and approved the final manuscript.

## CONFLICT OF INTEREST STATEMENT

The authors declare no conflict of interest.

## ETHICS STATEMENT

Not applicable.

## Data Availability

Not applicable.
